# Processing by MRE11 is involved in the sensitivity of subtelomeric regions to DNA double-strand breaks

**DOI:** 10.1093/nar/gkv714

**Published:** 2015-07-23

**Authors:** Keiko Muraki, Limei Han, Douglas Miller, John P. Murnane

**Affiliations:** Department of Radiation Oncology, University of California, San Francisco, 2340 Sutter St. San Francisco, CA 94143-1330, USA

## Abstract

The caps on the ends of chromosomes, called telomeres, keep the ends of chromosomes from appearing as DNA double-strand breaks (DSBs) and prevent chromosome fusion. However, subtelomeric regions are sensitive to DSBs, which in normal cells is responsible for ionizing radiation-induced cell senescence and protection against oncogene-induced replication stress, but promotes chromosome instability in cancer cells that lack cell cycle checkpoints. We have previously reported that I-*Sce*I endonuclease-induced DSBs near telomeres in a human cancer cell line are much more likely to generate large deletions and gross chromosome rearrangements (GCRs) than interstitial DSBs, but found no difference in the frequency of I-*Sce*I-induced small deletions at interstitial and subtelomeric DSBs. We now show that inhibition of MRE11 3′–5′ exonuclease activity with Mirin reduces the frequency of large deletions and GCRs at both interstitial and subtelomeric DSBs, but has little effect on the frequency of small deletions. We conclude that large deletions and GCRs are due to excessive processing of DSBs, while most small deletions occur during classical nonhomologous end joining (C-NHEJ). The sensitivity of subtelomeric regions to DSBs is therefore because they are prone to undergo excessive processing, and not because of a deficiency in C-NHEJ in subtelomeric regions.

## INTRODUCTION

DNA double-strand breaks (DSBs) from extracellular origins, such as ionizing radiation (IR) or chemotherapeutic drugs, or from intracellular origins, such as replication fork collapse or reactive oxygen species, are very hazardous to cells (1). If not repaired properly, DSBs can cause mutations and chromosome rearrangements, resulting in cancer or cell death. Thus, cells are equipped with multiple pathways for repair of DSBs. Classical nonhomologous end joining (C-NHEJ) is the primary mechanism for repairing DSBs in mammalian cells (1). C-NHEJ utilizes a complex containing the KU70 and KU86 proteins (KU70/86) to protect the ends of DSBs from nuclease digestion and to tether the ends together to facilitate rejoining (2). DSBs can also be repaired through pathways that involve the binding of the MRE11-RAD50-NBS1 (MRN) complex. After binding to the DSB, the MRN complex tethers the ends together and activates the Ataxia-Telangiectasia Mutated Protein (ATM), which in turn initiates the DNA damage response (3) and protects the ends of the DSB by recruiting the P53-Binding Protein 1 (53BP1) and MAD2 Mitotic Arrest Deficient-Like 2 (MAD2L2) proteins (4,5). The MRN/ATM pathway is involved in chromatin modification, which is required for C-NHEJ when DSBs occur in heterochromatin (6,7), or when C-NHEJ does not occur in a timely manner (2). The MRN/ATM pathway is also involved in the processing of DSBs, which is required for homologous recombination repair (HRR) and alternative NHEJ (A-NHEJ). The processing of DSBs results in the formation of a 3′ single-stranded overhang, which involves the nuclease activity of the Meiotic Recombination 11 Homolog (MRE11) and CtBP-Interacting Protein (CtIP) (8,9). The processing of DSBs by the nuclease activity of MRE11 can be independent of ATM (10–12). However, during late S and early G2 phase, ATM and Cyclin Dependent Kinase (CDK) function together in the activation of CtIP (13,14), which, like MRE11, contains nuclease activity used in processing DSBs (15). The processing of DSBs by MRE11 and CtIP is then followed by extensive resection of the 5′ ends of the DSB by the nuclease activity of Exonuclease 1 (EXOI) or the BLM-DNA2 complex (16–18). When processing occurs during late S and early G2 phase, DSB repair can occur through HRR, which repairs DSBs precisely by using the sister chromatid as a template for DNA synthesis. However, if HRR cannot occur, as is the case when processing occurs in G1 phase, the processed ends must be joined by A-NHEJ. A-NHEJ involves joining at sites with or without microhomology within the 3′ single-stranded overhangs, and is commonly involved in deletions, insertions, translocations, inversions, and other complex rearrangements (19–21). A-NHEJ can occur without the extensive resection of DSB ends that is required for HRR, as shown by the fact that A-NHEJ can occur in cells deficient in BRCA1, EXO1 or BLM1, which have a deficiency in HRR (22,23).

The choice of which pathway is used for DSB repair is tightly regulated, with the C-NHEJ pathway inhibiting the processing required by the HRR and A-NHEJ pathways, and the processing and resection required by the HRR pathway inhibiting the C-NHEJ pathway. The initial step in processing by MRE11 and CtIP is shared by both the HRR and A-NHEJ pathways (23). ATM protects DSB ends from processing by MRE11, so that a deficiency in ATM results in an increase in A-NHEJ (24). Similarly, the loss of protection due to a deficiency in KU70 or KU86 increases resection and promotes HRR and A-NHEJ, while a deficiency in end resection abolishes HRR and promotes repair by C-NHEJ (23,25–28). Conversely, the 3′ single-stranded overhangs resulting from processing and resection inhibit C-NHEJ, so that once processing is initiated, DSBs must be repaired by either HRR or A-NHEJ (29). To overcome the inhibition of HRR, the combined nuclease activity of MRE11 and CtIP promotes the release of the KU70/86 and MRN complexes from DSBs (8,9). Similarly, the removal of 53BP1 by BRCA1 is required for HRR (30). Although the absence of the nuclease activity of CtIP strongly diminishes HRR foci formation in G_2_ phase in response to ionizing radiation (15), ATM and the nuclease activity of CtIP are not required for HRR for I-*Sce*I-induced DSBs (15,31,32). Therefore, the processing required for HRR for DSBs generated by I-*Sce*I endonuclease can occur without MRE11 or the nuclease activity of CtIP.

Most DSBs within euchromatin are repaired by the C-NHEJ pathway, which occurs by fast kinetics and is independent of ATM. Conversely, slow repair is ATM dependent and includes HRR as well as DSBs that are difficult to repair, including multiply damaged sites (28,33) and DSBs in heterochromatin (6,7). DSBs occurring near telomeres are also inefficiently repaired. Telomeres are caps on the ends of chromosomes that distinguish chromosome ends from DSBs, and thus prevent chromosome end-to-end fusion and genomic instability (34,35). Mammalian telomeres are composed of thousands of tandem TTAGGG repeats with a 3′ single-stranded overhang, and a variety of telomere-associated proteins called the shelterin complex (35). IR-induced DSBs that occur near telomeres can persist for long periods of time, and are associated with replicative senescence in mammalian cells in culture and *in vivo* (36,37). However, in mouse embryonic stem cells and cancer cells, which do not initiate replicative senescence, DSBs near telomeres result in telomere loss and genomic instability (38–40). This observation led to the proposal that the sensitivity of telomeric regions to DSBs contributes to the high rate of spontaneous telomere loss that promotes chromosome instability in human cancer (41).

We have previously shown that DSBs occurring within subtelomeric regions are much more likely to generate large deletions and GCRs, and that these deletions are much larger than at interstitial DSBs, demonstrating that extensive degradation is a common feature at DSBs near telomeres (40,42). To analyze the mechanism responsible for this sensitivity of subtelomeric regions to DSBs, we have utilized cell clones that contain a variety of reporter constructs that allow us to compare the types of mutations occurring at I-*Sce*I endonuclease-induced DSBs at interstitial and subtelomeric sites. The use of I-*Sce*I endonuclease allows for the targeting of DSBs to specific locations, and has been used extensively to study the mechanisms of DSB repair (26,43–47). The vast majority of I-*Sce*I-induced DSBs at interstitial sites are precisely rejoined by C-NHEJ (48), although a small percentage result in mutations (26,43–47), several of which are detected by our reporter assays, including large deletions, gross chromosome rearrangements (GCRs), small deletions, and deletions resulting from the joining of the distal ends of two DSBs located in close proximity on the same chromosome (distal NHEJ). Our initial study used these assays to investigated the role of ATM in the formation of mutations during DSB repair (49). ATM was chosen for analysis because ATM is inhibited by the shelterin protein TRF2 (50). This could impact DSB repair near telomeres, because subtelomeric regions are composed of heterochromatin (51) and ATM is required for DSB repair in heterochromatin (6,7). However, our results showed that the inhibition of ATM caused a further increase in the high frequency of large deletions at subtelomeric DSBs, and decreased the frequency of GCRs, small deletions, and distal NHEJ (49). ATM is therefore functional near telomeres, and actually suppresses the processing and resection involved in the formation of large deletions, despite the fact that some functions of ATM are inhibited at telomeres (52). We therefore proposed an alternative model in which the sensitivity of subtelomeric regions to DSBs results from excessive processing of DSBs, possibly because DSBs in subtelomeric regions are mistaken for telomeres (49), which are processed by the nucleases MRE11 (53–55) and Apollo (56,57).

Our current study addresses our hypothesis that the sensitivity of DSBs near telomeres is a result of excessive processing by investigating the role of MRE11, which is one of the nucleases involved in the processing of DSBs (8,9) and telomeres (53–55), and has been associated with excessive processing of unprotected DSBs (24). Our results show that the inhibition of the 3′–5′ exonuclease activity of MRE11 with Mirin partially inhibits the formation large deletions and GCRs at both interstitial and subtelomeric DSBs, although to a greater degree at subtelomeric DSBs. MRE11 therefore contributes to the excessive processing of DSBs leading to large deletions and GCRs, although other nucleases, such as CtIP and EXO1 that are also involved in processing of unprotected DSBs (4,5,58) are also likely to be involved. Conversely, we found that the inhibition of the 3′–5′ exonuclease activity of MRE11 with Mirin has little effect on the formation of small deletions at either interstitial or subtelomeric DSBs, suggesting that most small deletions occur through C-NHEJ, which does not require processing by MRE11. Because the frequency of small deletions is the same at interstitial and subtelomeric DSBs, we conclude that C-NHEJ is not deficient near telomeres. Our combined results support a mechanism in which the increased frequency of large deletions and GCRs at subtelomeric DSBs is a result of excessive processing of DSBs and is not due to a defect in DSB repair by C-NHEJ.

## MATERIALS AND METHODS

### Plasmids

The pEJ5-GFP plasmid, pGFP-ISceI plasmid and the pDsRed plasmid have been previously used to monitor the frequency of distal NHEJ, large deletions and GCRs, respectively (26,42,49).

### Cell lines

All of the cell lines used in this study were derived from clone B3–4 of the EJ-30 human bladder cell carcinoma cell line. EJ-30 is a subclone of the EJ human colon cancer cell line, which is also called MGH-U1 (59). The cells were grown in MEM alpha media (UCSF Cell Culture Facility) supplemented with 5% fetal calf serum (Invitrogen-Gibco), 5% newborn calf serum (Invitrogen-Gibco), 1 mM *L*-glutamine (Invitrogen-Gibco), and were propagated at 37°C in humidified incubators.

The GFP-7F1 and GFP-6D1 cell clones containing the pGFP-ISceI plasmid integrated at interstitial and telomeric sites, respectively, were previously used to investigate the frequency of large deletions (42,49). The EJ5–7F2 and EJ5–6J8 cell clones containing the pEJ5-GFP plasmid integrated at interstitial and telomeric sites, respectively, and the pDsRed plasmid integrated at interstitial sites, were previously used to investigate the frequency of distal NHEJ and GCRs (42,49).

### Generation of I-SceI-induced DSBs

Packaging of the pQCXIH and pQCXIH-ISceI retroviral vectors and infection of cell cultures was performed as previously described (40). The selection for cells infected with pQCXIH-ISceI was achieved by growth in medium containing 50 μg/ml hygromycin (Sigma) for 14 or 15 days, with medium changes every 2 days to allow for expression of I-*Sce*I endonuclease and the generation of DSBs. After 13 days, the cells were trypsinized and replated. After an additional 1 or 2 days, the cells were trypsinized again, pooled, and either analyzed for the frequency of GFP-positive and DsRed-positive cells, or used for isolation of genomic DNA for analysis of small deletions.

### shRNA-mediated knockdown of gene expression

The effect of MRE11 on our assays was investigated using shRNA-mediated knockdown of MRE11 expression in each of our clones. These cell clones were isolated just prior to performing our experiments using Blasticidin selection for the pSIREN-Blast plasmid (Clontech, pSIREN-RetroQ with a Blastocidin gene instead of a puro gene) containing the shRNA insert. Individual clones were then analyzed for the extent of knockdown by qPCR just prior to performing the experiments, as well as by western blot analysis and qPCR after performing the experiments. The sequence used for MRE11 knockdown is 5′-GATGCCATTGAGGAATAAG-3′ (11), and the primers for qPCR are MRE11-F; 5′-GCCTTCCCGAAATGTCACTA-3′ and MRE11-R; 5′-TTCAAAATCAACCCCTTTCG-3′ (60). The sequence used for Luciferase knockdown as a control is 5′-CGTACGCGGAATACTTCGA-3′ (58). After transfection with pSiren-RetroQ-Blasticidin, cells were selected with Blasticidin for 12 days, transferred into new culture flasks, and at day 13 the cells were collected and frozen in multiple aliquots. After thawing and culturing for two to three days, one aliquot was used for q-PCR, one for western blot analysis, and the other aliquots were used for analysis in our various assays for large deletions, GCRs, small deletions and distal NHEJ. shRNA-mediated knockdown of ATM was performed as described (42,49).

### Treatment with Mirin

Treatment of cells with 20 μM Mirin (Sigma-Aldrich), an inhibitor of MRE11 (7,61–63), began one day after infection with the pQCXIH or pQCXIH-ISceI retroviruses and continued for 14 or 15 days prior to cell analysis.

### Quantitative real-time PCR

Quantitative real-time PCR (qPCR) analysis to analyze knockdown efficiency was done as previously described (42,49), using a StepOnePlus Real-Time PCR machine (Applied Biosystems). A mixture of cDNA from cell clones EDS-6J8, EDS-7F2, GFP-6D1 and GFP-7F1 that was undiluted, diluted 4X, 16X, and 64X, was used as a standard. The level of expression of the housekeeping gene GAPDH was also analyzed in each sample to control for the efficiency of PCR in each sample. The knockdown efficiency of ATM and MRE11 was calculated by comparing the expression level of the ATM, MRE11 and GAPDH genes in cell cultures with and without the shRNA for ATM or MRE11. The expression level of the ATM, MRE11 and GAPDH genes were calculated by absolute quantification relative to the standard curve using the Standard Curve Method with the SDS software provided by the manufacturer (Applied Biosystems). The primer sequences used for qPCR for analysis of knockdown of ATM (42,49), MRE11 (60) and GAPDH (42,49) have been previously published.

### Western blot analysis

Cells were harvested by trypsinization, and resuspended in urea lysis buffer (9 M urea, 150 mM β-mercaptoethanol, 75 mM Tris, pH 7.4). The lysate were sheared by sonication, centrifuged at 15000 rpm for 30 min, and the soluble fractions were collected. Protein concentration was determined using Quick Start^TM^ Bradford 1x Dye Reagent (BioRad) and equal amounts of proteins were separated on 7.5% Tris-HCl SDS-polyacrylamid gels and transferred to Immobilon-P membrane (IPVH00010; EMD Millipore). The membrane was blocked in 5% milk in TBS with 0.02% Tween-20 and incubated with anti-ATM antibody (NB100–309; Novus), anti-ATM (phosphor S1981) antibody (ab81292; abcam), anti-MRE11 antibody (NB100–142; Novus), and anti-GAPDH antibody (#5174; Cell Signaling). ECL^TM^ Anti-rabbit IgG, horseradish peroxidase linked whole antibody (NA934V; GE Healthcare) and ECL^TM^ Anti-mouse IgG, horseradish peroxidase linked whole antibody (NA931V; GE Healthcare) were used as secondary antibodies. ECL western blotting substrate (Pierce) was used for detection by FluorChem^TM^ E (Protein Simple).

### Analysis of large deletions

The frequency of GFP^+^ cells was determined using a Cellometer Vision (Nexelcom), as previously described (42,49). The cells were first trypsinized and 20 μl of growth medium containing approximately 1 × 10^4^ cells was aliquoted into a counting chamber slide (Nexelcom). Two counting chambers were used for each sample, with each chamber being counted two times. All samples were analyzed in triplicate. Error bars represent standard deviation of experiments that were conducted three times.

### Flow cytometry of GFP-positive and DsRed-positive cells

The analysis of the frequency of GFP-positive and DsRed-positive cells was performed using an Accuri C6 Flow Cytometer (BD Biosciences), as previously described (42,49). Cells were trypsinized and removed from the plate, an equal volume of growth medium was added, after which the cells were counted and pelleted. The cells were then resuspended in 10 ml of ice-cold Dulbecco's PBS (w/o Ca or Mg) containing 100 μg/ml Proteinase K (Sigma) by vigorous pipeting with a fine bore plastic pipet. The cells were then incubated 10 min on ice, pipeting twice more during the incubation. This treatment with Proteinase K is necessary with the EJ-30 cell line to keep the cells from aggregating. Following the incubation with proteinase K, 2 ml of Dulbecco's PBS (w/o Ca or Mg) containing 1% BSA (Sigma) was added to block further digestion. The cells were then pelleted and resuspended in Dulbecco's PBS (w/o Ca or Mg) at approximately 1 × 10^6^ cells/ml for analysis by flow cytometry. Approximately 1 × 10^6^ cells were counted for each sample. All samples were analyzed in triplicate. Error bars represent standard deviation of experiments that were conducted three or more times.

### Analysis of small deletions

The frequency of small deletions at an I-*Sce*I-induced DSB in the integrated pEJ5-GFP plasmid was determined as previously described (42,49), by first generating PCR products spanning one of the I-*Sce*I sites using genomic DNA isolated from the pooled population of cells infected with pQCXIH-ISceI and selected with hygromycin for 14 or 15 days (see Figure 4A and Supplementary Figure S5). The PCR products were then digested with I-*Sce*I endonuclease and run on agarose gels as previously described (42,49). PCR was performed using Taq 2X Master Mix (New England Biolabs) and primers GFP-1 (5′-GCGGGGTTCGGCTTCTGG-3′) and GFP-3 (5′-CGCTTCCATTGCTCAGCGG-3′) for Figure 4, and GFP-1 and GFP-2 (5′-TCGGGCATGGCGGACTTG-3′) for Supplementary Figure S5. PCR involved 94°C for 2 min, then 40 cycles of 94°C for 30 s, 62°C for 30 s, and 72°C for 30 s. 25 μl of the PCR product was then digested with 20 units of I-*Sce*I endonuclease at 37°C overnight, and the products were run on 4% agarose gels. After staining with ethidium bromide, digital images were analyzed using Image J software (http://www.versiontracker.com/dyn/moreinfo/macosx/37303) to calculate the intensity of the bands. The fraction of cells containing small deletions (SD) at the I-*Sce*I site was determined by dividing the intensity of the uncut band (UC) by the combined intensity of the cut (C) and uncut bands. The values for small deletions were then corrected for the fraction of cells that had large deletions or NHEJ, because these cells would not produce a PCR product, and would therefore cause an overestimation of the fraction of cells containing small deletions. The fraction of cells with small deletions therefore involves multiplying the fraction of uncut PCR product by 1 minus the fraction of cells with large deletions (LD), as determined in our large deletion assay, and by 1 minus the fraction of cells with distal NHEJ, as determined by our distal NHEJ assay. The final equation for the fraction of cells with small deletions is therefore: SD = UC/(UC+C) x (1 - LD)(1 - distal NHEJ) for Figure 4 and SD = UC/(UC+C) x (1 - LD) for Supplementary Figure S5. Although inversions of the fragment between the two I-*Sce*I sites would also prevent small deletions, this was not corrected for because the frequency of these events is too low to significantly affect our results (64). The validity of this correction was previously demonstrated by the analysis of the frequency of small deletions in 100 individual subclones selected at random (40). All samples were analyzed in triplicate. Error bars represent standard deviation of experiments that were conducted three or more times.

## RESULTS

### Investigating the role of MRE11 in the formation of mutations during DSB repair

**Figure 1.**
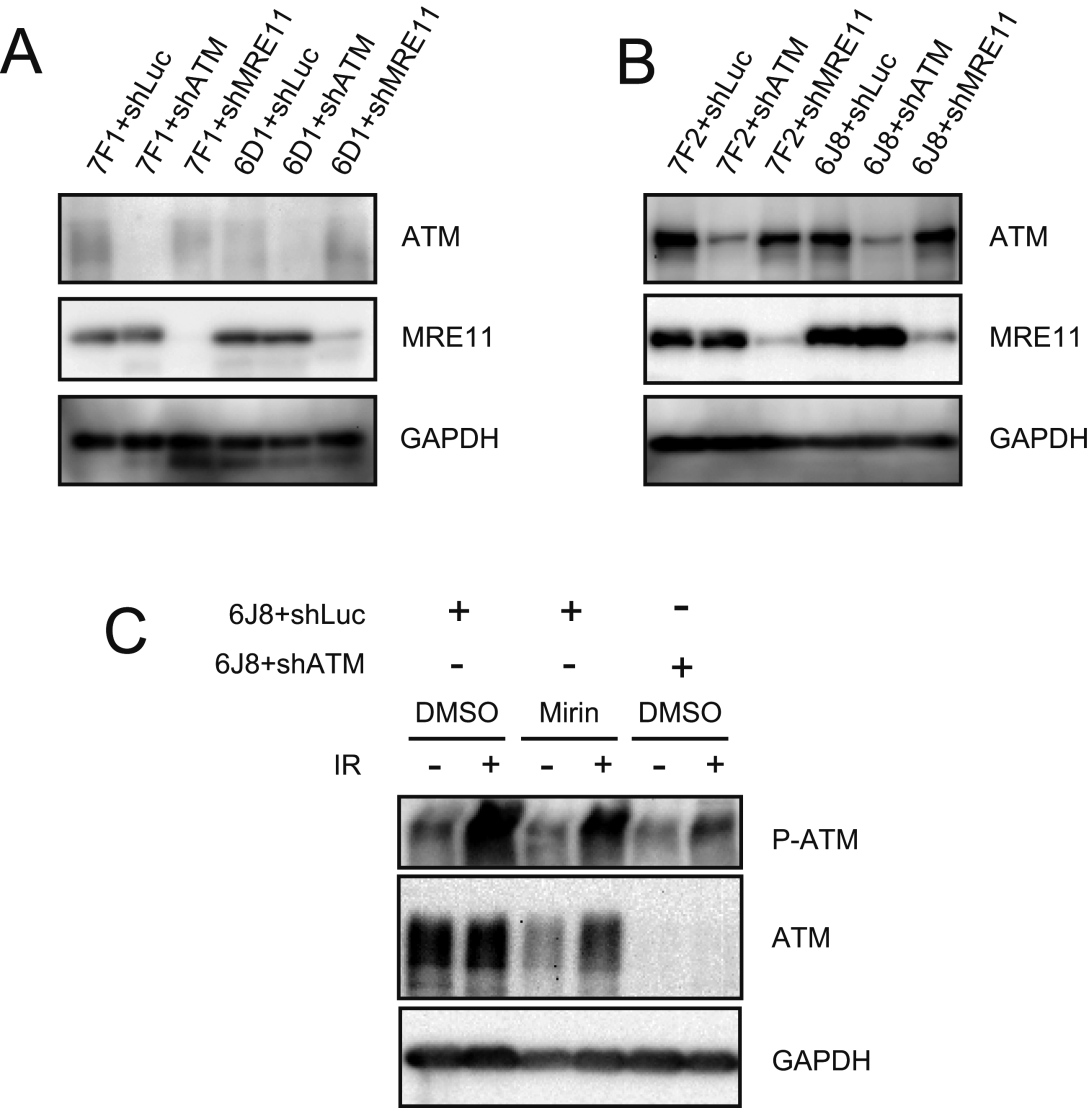
Western blot analysis of the extent of knockdown MRE11 and ATM, and the lack of effect of Mirin activation of ATM. The extent of shRNA-mediated knockdown of MRE11 and ATM are shown for (**A**) clones GFP-7F1 and GFP-6D1 and (**B**) clones EDS-7F2 and EDS-6J8. The extent of knockdown was determined by analyzing the intensity of the ATM and MRE11 bands relative to the intensity of the loading control GAPDH bands (see Table 1). (**C**) The effect of Mirin on activation of ATM was determined by analysis of phosphorylation of ATM in response to ionizing radiation. Cultures treated with DMSO alone, 20 μM Mirin, or knockdown of ATM were analyzed by western blot 30 min after exposure to 10 Gy of ionizing radiation.

To address our hypothesis that excessive processing is the reason that subtelomeric DSBs are highly prone to large deletions and GCRs, we investigated the role of MRE11 in the formation of mutations at interstitial and subtelomeric DSBs. MRE11 was selected, because it is one of several nucleases that are known to be involved in the processing of DSBs (8,9) and telomeres (53–55,65). MRE11 has also been found to be involved in excessive processing of unprotected DSBs (24), although other nucleases can also contribute to excessive processing of DSBs (4,5,58). Studies involving MRE11 are complicated by the multiple functions of MRE11 and the MRN complex, which include tethering DNA to facilitate DSB repair, activation of ATM and the DNA damage response, and the processing of DSBs through its nuclease activity in combination with CtIP (3). The inhibition of MRE11 in our study was accomplished by either shRNA-mediated knockdown or treatment with Mirin. The extent of knockdown of MRE11 following stable integration of the shMRE11 vector as measured by quantitative real-time PCR (qPCR) was 77% in clone GFP-7F1 and 74% in clone GFP-6D1 (Table 1), while the extent of knockdown by western blot analysis was 92% in clone GFP-7F1 and 79% in clone GFP-6D1 (Figure 1A, Table 1). The extent of knockdown of ATM by shATM as measured by qPCR was 81% in clone GFP-7F1 and 76% in clone GFP-6D1 (Table 1). The extent of knockdown of ATM by western blot analysis was estimated to be approximately 90% in clone GFP-7F1 and 70% in clone GFP-6D1 (Figure 1B, Table 1). However, due to the diffuse nature of the ATM bands, which was reproducible, it was not possible to determine the exact extent of knockdown, although it is clear that a significant knockdown of ATM was achieved. Similar diffuse ATM bands have been observed by western blot in other cell lines (7).

**Table 1.** Extent of shRNA-mediated knockdown of MRE11 and ATM as determined by quantitative real time PCR (qPCR) and western blot analysis

		GFP-7F1	GFP-6D1	EDS-7F2	EDS-6J8
shATM	qPCR	81%	76%	89%	71%
	Western blot	90%^a^	70%^a^	85%	77%
shMRE11	qPCR	77%	74%	80%	80%
	Western blot	92%	79%	85%	77%

^a^These are approximations due to the diffuse nature of the ATM bands.

*In vitro*, Mirin can independently inhibit both the nuclease activity of MRE11 and the ability of MRE11 to activate ATM, without inhibiting the tethering function of the MRN complex (61,62). However, at the concentrations used in cells in culture, Mirin inhibits only the 3′–5′ exonuclease activity of MRE11 without affecting its ability to activate ATM (7,63). Shibata *et al*. showed that Mirin and PFM39, a compound derived from Mirin that also binds and inhibits the 3′–5′ exonuclease activity of MRE11, do not inhibit the ability of MRE11 to activate ATM (7). This was achieved by demonstrating that following ionizing radiation, Mirin does not prevent the phosphorylation of ATM, and PFM39 does not prevent the formation of phosphorylated KAP-1 foci. Consistent with the work of Shibata *et al*. (7), we also found that Mirin did not inhibit the activation of ATM at the concentration used in our cell clones (Figure 1C). Shibata *et al*. also demonstrated the specificity of Mirin for MRE11 by x-ray crystallography and by showing that the inhibition of processing of DSBs by PFM39 was not observed in ATLD cells deficient in MRE11. We therefore felt confident in using Mirin to address the role of MRE11 3′–5′ exonuclease activity in DSB repair.

### Processing by MRE11 is involved in the formation of some large deletions at interstitial and subtelomeric DSBs

**Figure 2.**
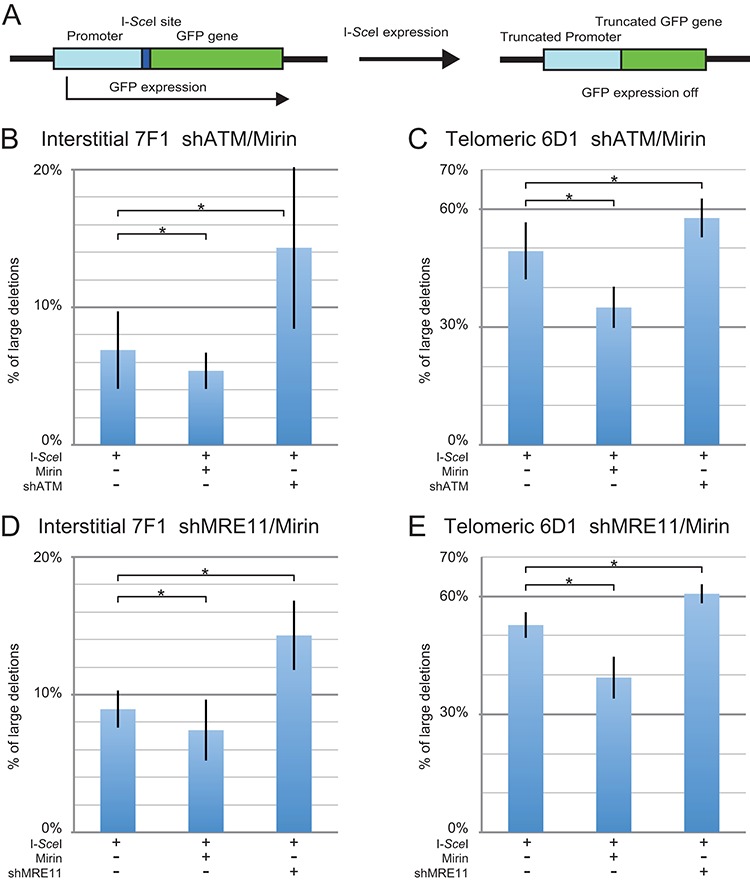
The effect of inhibition of MRE11 and ATM on large deletions at interstitial and subtelomeric DSBs. (**A**) Cell clones containing the pGFP-ISceI plasmid integrated at an interstitial (GFP-7F1) or telomeric (GFP-6D1) site were used for analysis of large deletions. The GFP gene in the integrated pGFP-ISceI plasmid is inactivated following large deletions of more than 28 bp at the I-*Sce*I-induced DSB. The frequency of large deletions (GFP-negative cells) at the I-*Sce*I-induced DSB was determined for clone GFP-7F1 (**B, D**) and clone GFP-6D1 (**C, E**) following infection with the pQCXIH-ISceI retrovirus vector and selection with hygromycin for 14 days. Large deletions were analyzed following (**B, C**) treatment with Mirin or knockdown of ATM (shATM), or (**D, E**) treatment with Mirin or knockdown of MRE11 (shMRE11). Control cultures for knockdown of ATM or MRE11 were treated with shRNA-mediated knockdown of luciferase, while control cultures for Mirin were treated with DMSO. The values shown in the graph represent the average of three independent experiments, each done in triplicate. Error bars represent the standard deviation of the three separate experiments. Statistical significance for comparisons between the indicated values (horizontal lines) was determined using the two-tailed Student's *t*-test, and an asterisk indicates statistically significant values of 0.05 or less.

The assay used for these studies involves clones of the EJ-30 human bladder cell carcinoma cell line that contain the pGFP-ISceI plasmid integrated at interstitial or telomeric sites (Figure 2A), as we have previously reported (42,49). In this assay, DSBs are introduced at an I-*Sce*I recognition site that is located between the green fluorescent protein (GFP) coding sequence and its chicken β-actin promoter. The degradation of more than 28 bp at the I-*Sce*I-induced DSB (large deletions) results in the loss of GFP expression due to the inactivation of either the promoter or the GFP gene. The I-*Sce*I-induced DSBs in our system are introduced by infection with the pQCXIH-ISceI retrovirus and selection with hygromycin, so that all of the cells constitutively express I-*Sce*I endonuclease. As a control, we also infected the cells with the pQCXIH retrovirus without the gene for I-*Sce*I endonuclease. Importantly, although the size of the deletions was not analyzed in our current study, the pGFP-ISceI assay used here demonstrates the same frequency of large deletions at interstitial and subtelomeric DSBs that was observed in our earlier more in-depth DNA analysis, which showed that the I-*Sce*I-induced large deletions at subtelomeric DSBs are much larger than those at interstitial DSBs, commonly extending more than several kilobases (38,40). This sensitivity of subtelomeric regions to DSBs is not dependent on the integration site, in that we have seen this high frequency of large deletions in multiple clones of mouse embryonic stem cells (39,66) and human tumor cell clones (38,40,67) with different telomeric integration sites. In addition to large deletions, a variety of other events can also result in the loss of expression of the GFP gene in this assay, including GCRs, the silencing of the GFP gene due to telomere position effect, and *de novo* telomere addition at the DSB (chromosome healing). However, our previous studies involving Southern blot analysis and DNA sequencing demonstrated that large deletions account for 95% of the rearrangements that would result in inactivation of the GFP gene in our current assay (40).

The expression of I-*Sce*I endonuclease in clone GFP-7F1, which has an interstitial DSB, resulted in an average 7.7% of the cells becoming GFP negative (Table 2) when combining the six experiments conducted for Figure 2B and D, meaning that 7.7% of the cells experienced large deletions. The inhibition of MRE11 with Mirin in clone GFP-7F1 caused an average 19% relative decrease in the frequency of I-*Sce*I-induced large deletions (Table 3) when combining the six experiments conducted for Figures 1D and 2B. Therefore, MRE11 is involved in the formation of large deletions at some interstitial DSBs.

**Table 2.** The frequency of mutations detected by our various assays at interstitial and telomeric DSBs, and the ratio of the frequency of telomeric to interstitial mutations

Assay	Interstitial^a^	Telomeric^a^	Ratio Tel/Int	Corr Ratio^b^
Large deletions	7.7 ± 2.5	51.1 ± 3.9	6.6	8.6
GCRs	0.0052 ± 0.0015	0.086 ± 0.054	17	20.6
Small deletions	24.2 ± 3.7	18.5 ± 3.0	0.77	0.99
Distal NHEJ	7.8 ± 1.7	2.0 ± 0.5	0.26	0.34

^a^The values shown are the average of the control cultures treated with I-*Sce*I alone from the two different experiments (Figs. B and D for interstitial DSBs, and Figs. C and E for telomeric DSBs.

^b^Values corrected for loss of the plasmid sequences due to large deletions, averaged over the duration of experiment, which is the final frequency of large deletions divided by 2.

Interstitial: 7.7%/2 = 3.9% average, correction factor 100/96.1 = 1.04.

Telomeric: 51%/2 = 25.5% average, correction factor 100/74.5 = 1.34.

**Table 3.** Effect of Mirin on the relative (Rel) and actual (Act) number of large deletions, GCRs, distal NHEJ, and small deletions in cell clones with an interstitial (Int) or telomeric (Tel) DSB

	Rel decrease (%)		Act decrease (%)	
Assay	Int^a^	Tel^a^	Int^a^	Tel^a^
Large deletions	19	27	1.5	13.8
GCRs	50	51	0.0026	0.044
Small deletions	9.4	−16	2.3	−3.0
Distal NHEJ	32	−23	2.4	−0.47

^a^The values shown are the average of the cultures treated with Mirin from the two different experiments (Figs. B and D for interstitial DSBs, and Figs. C and E for telomeric DSBs.

In clone GFP-6D1, which has a subtelomeric DSB, the expression of I-*Sce*I resulted in an average of 51.1% of the cells becoming GFP negative (Table 2) when combining the six experiments conducted for Figure 2C and E, meaning that 51.1% of the cells experienced large deletions. The inhibition of MRE11 with Mirin caused an average 27% relative decrease in the frequency of large deletions (Table 3) when combining the six experiments conducted for Figure 2C and E. The relative decrease in large deletions caused by Mirin was therefore higher at subtelomeric DSBs than at interstitial DSBs (27 and 19%, respectively). Furthermore, the actual decrease in large deletions was much less at interstitial DSBs than at subtelomeric DSBs (1.5 and 14%, respectively) (Table 3), and therefore MRE11 is much more likely to generate large deletions at subtelomeric DSBs than at interstitial DSBs. However, because Mirin did not prevent most large deletions, it appears that other nucleases are also responsible for the formation of large deletions at interstitial and subtelomeric DSBs. Regardless, the effects of Mirin on large deletions are consistent with a role for excessive processing in the sensitivity of subtelomeric regions to DSBs.

### The knockdown of MRE11 has the opposite effect of Mirin on large deletion formation

We investigated the effect of knockdown of MRE11 on large deletions to determine how the loss of all MRE11 functions compares with the inhibition of MRE11 3′–5′ exonuclease activity with Mirin. In addition to its role in processing of DSBs, MRE11 is involved in the activation of ATM, which is independent of the nuclease activity of MRE11. We previously showed that knockdown of ATM or inhibition of ATM kinase activity with KU55933 caused an increase in large deletions (49). Consistent with our previous report (49), in clone GFP-7F1 with an interstitial DSB, ATM knockdown caused a 108% relative increase (from 6.9% to 14.3%) in I-*Sce*I-induced large deletions (Figure 2B and Supplementary Figure S1B). Similarly, the knockdown of MRE11 in clone GFP-7F1 caused a 60% relative increase (from 8.9% to 14.3%) in large deletions (Figure 2D and Supplementary Figure S1D). Like the knockdown of ATM, the knockdown of MRE11 therefore causes the opposite effect on large deletion formation as that caused by the inhibition of MRE11 with Mirin. This similarity in the effects of knockdown of MRE11 and ATM on large deletions is consistent with the role of MRE11 in the activation of ATM, and the importance of ATM in preventing the formation of large deletions at interstitial DSBs (49). In addition, the difference in the response to knockdown of MRE11 and the inhibition of MRE11 with Mirin further suggests that the effects of Mirin are a result of the inhibition of MRE11 3′–5′ exonuclease activity, and not MRE11-mediated activation of ATM. The increase in large deletions resulting from inhibition of ATM could result from either a loss of ATM-dependent DSB repair, or a loss of protection of DSBs, which is ATM dependent (4). A loss of DSB repair is not likely, because HRR resulting from I-*Sce*I-induced DSBs does not require ATM (15,31,32). ATM is required for C-NHEJ for DSBs occurring within heterochromatin (6,7), however, the high uniform level of expression of the GFP gene in clone GFP-7F1 (Supplementary Figure S2) indicates that the pGFP-ISceI plasmid is not integrated within heterochromatin. Furthermore, we have previously shown that ATM knockdown did not affect GCRs, small deletions, or distal NHEJ at interstitial DSBs (49). The increase in I-*Sce*I-induced large deletions in clone GFP-7F1 is therefore most likely due to the loss of ATM-dependent protection of DSBs.

Consistent with our earlier report (49), the knockdown of ATM in clone GFP-6D1 with a subtelomeric DSB caused a 17% relative increase (from 49% to 58%) in I-*Sce*I-induced large deletions (Figure 2C and Supplementary Figure S1C). Therefore, ATM is also involved in preventing the formation of large deletions at subtelomeric DSBs, although much less so than at interstitial DSBs. Similarly, the knockdown of MRE11 in clone GFP-6D1 caused a 15% relative increase (from 53% to 61%) in the frequency of large deletions (Figure 2E and Supplementary Figure S1E). Importantly, the increase in I-*Sce*I-induced large deletions caused by knockdown of MRE11 is again similar to the increase in large deletions caused by knockdown of ATM at both interstitial and subtelomeric DSBs, suggesting that the effect of knockdown of MRE11 is a result of the failure to activate ATM. Consistent with this conclusion, the effects of knockdown of ATM on large deletions and GCRs are also similar to the inhibition of ATM kinase activity using KU55933 (49). Importantly, our results show that at both interstitial and subtelomeric DSBs, Mirin has the opposite effect of knockdown of MRE11 or ATM, demonstrating that as previously reported in other studies (7,63), the effects of Mirin in our cells is a result of the inhibition of the 3′–5′ exonuclease activity of MRE11, and not by inhibiting the activation of ATM. As with interstitial DSBs, the increase in the frequency of large deletions at subtelomeric DSBs resulting from the inhibition of ATM could result from either the loss of ATM-dependent protection or the requirement for ATM for repair by C-NHEJ. However, unlike with the interstitial DSB in clone GFP-7F1, the repair of subtelomeric DSBs in clone GFP-6D1 might require ATM because subtelomeric regions are composed of heterochromatin (51), and repair of DSBs occurring within heterochromatin is ATM dependent (6,7). The partial suppression of the GFP gene in clone GFP-6D1 suggests the presence of telomere position effect, which is associated with subtelomeric heterochromatin (Supplementary Figure S2). Additional evidence that ATM is required for repair of DSBs near telomeres is provided by the demonstration that the chromosome fusions resulting from a deficiency in TRF2 occurs by C-NHEJ that is ATM dependent (68).

### MRE11 is involved in the formation of GCRs at both interstitial and subtelomeric DSBs

**Figure 3.**
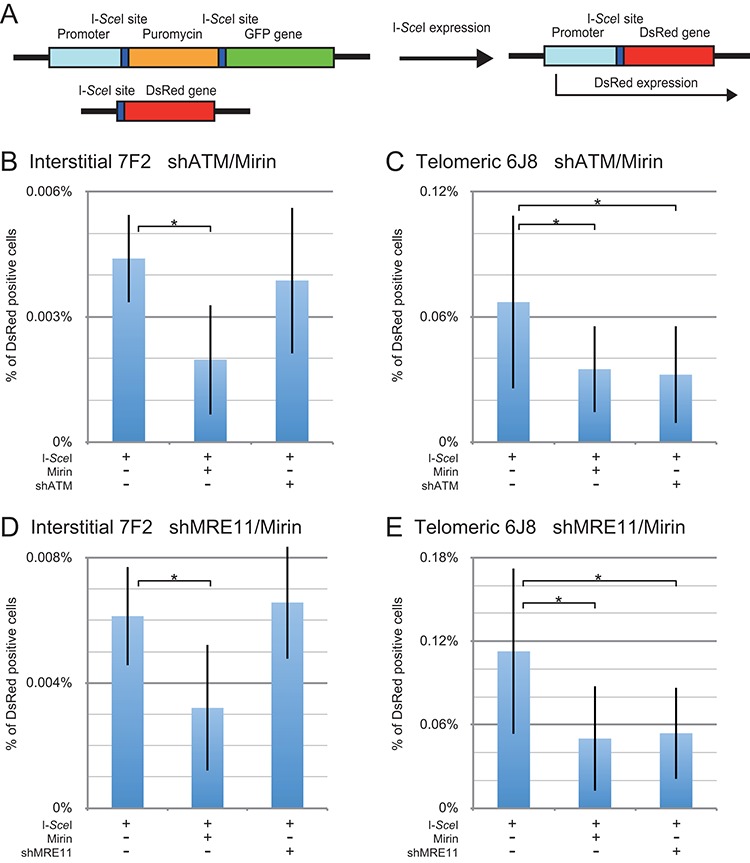
The effect of inhibition of MRE11 and ATM on GCRs at interstitial and subtelomeric DSBs. (**A**) The analysis of GCRs was performed using cell clones that contain the pEJ5-GFP plasmid integrated at an interstitial (EDS-7F2) or telomeric (EDS-6J8) site, and a pDsRed-ISceI plasmid integrated at an interstitial site. The DsRed gene in the pDsRed-ISceI plasmid is initially inactive due to the lack of a promoter, but is activated following NHEJ between the I-*Sce*I-induced DSBs in the pEJ5-GFP and pDsRed-ISceI plasmids. The frequency of GCRs (DsRed-positive cells) at the I-*Sce*I-induced DSB was determined for clone EDS-7F2 (**B, D**) and clone EDS-6J8 (**C, E**) following infection with the pQCXIH-ISceI retrovirus vector and selection with hygromycin for 14 days for EDS-7F2 and 15 days for EDS-6J8. GCRs were analyzed following (**B, C**) treatment with Mirin or knockdown of ATM (shATM), or (**D, E**) treatment with Mirin or knockdown of MRE11 (shMRE11). Control cultures for knockdown of ATM or MRE11 were treated with shRNA-mediated knockdown of luciferase, while control cultures for Mirin were treated with DMSO. The values shown in the graph represent the average of more than three independent experiments, each done in triplicate (see Supplementary Figures S3 and S4, and Table S1 for raw data). Error bars represent the standard deviation of the more than three separate experiments. Statistical significance for comparisons between the indicated values (horizontal lines) was determined using the two-tailed Student's *t*-test, and an asterisk indicates statistically significant values of 0.05 or less.

The involvement of MRE11 in the formation of GCRs was analyzed using our assay in which the relative frequency of GCRs is determined through the activation of the DsRed gene (Figure 3). We previously used this assay to demonstrate an increased frequency of GCRs at subtelomeric DSBs (49). The DsRed gene is initially inactive due to the absence of a transcriptional promoter, but is activated when the I-*Sce*I-induced DSB at the 3′ end of the chicken β-actin promoter in the pEJ5-GFP plasmid is joined with the I-*Sce*I-induced DSB at the 5′ end of the DsRed gene in the pDsRed-ISceI plasmid, which is integrated at a separate location (Figure 3A). Importantly, this assay only detects GCRs that occur without extensive degradation at the DSBs adjacent to the chicken β-actin promoter and DsRed gene. Our previous studies have shown that most large deletions at DSBs near telomeres result in the loss of the telomere (38) and therefore will also result in GCRs, although these GCRs are not detected by our GCR assay. As a result, an increase in degradation at the DSB will result in a decrease in the frequency of GCRs detected by our GCR assay, but not necessarily a decrease in actual GCRs. The major difference between our GCR assay and our large deletion assay is therefore the size of the deletions involved in the GCRs. Regardless, our GCR assay serves as a useful assay in combination with our other assays to establish the relative importance of various proteins in the formation of GCRs.

Our previous study showed very low levels of GCRs following the introduction of I-*Sce*I-induced DSBs in cell clones with an interstitial pEJ5-GFP plasmid and an interstitial pDsRed-ISceI plasmid (49). This low frequency of GCRs was similar in four different clones with the pDsRed-ISceI plasmid integrated at different random locations, consistent with the low frequency of GCRs detected by other studies that monitored GCRs occurring between two interstitial I-*Sce*I-induced DSBs (58,69–71). In contrast, the introduction of I-*Sce*I-induced DSBs in cell clones with a telomeric pEJ5-GFP plasmid and an interstitial pDsRed-ISceI plasmid resulted in high frequencies of GCRs (49). This high frequency of GCRs was observed in six clones with the pDsRed-ISceI plasmid integrated at different random locations, demonstrating that this difference in the frequency of GCRs is not due to the integration site of the pDsRed-ISceI plasmid. Two of these clones containing a single copy of the pDsRed-ISceI plasmid, one with the pEJ5-GFP plasmid at an interstitial site (EDS-7F2), and one with the pEJ5-GFP plasmid at a telomeric site (EDS-6J8), were used in our current studies. An example of a typical FACs analysis demonstrating I-*Sce*I-induced DsRed- and GFP-positive cells generated from clones EDS-7F2 and EDS-6J8 has been previously published (49).

Consistent with our earlier study (49), in clone EDS-7F2 with two interstitial I-*Sce*I-induced DSBs (one in pEJ5-GFP and one in pDsRed-ISceI), the frequency of I-*Sce*I-induced GCRs is very low, with an average of 0.0052% (Table 2) when combining the eight experiments conducted for Figure 3B and D. The inhibition of MRE11 with Mirin caused an average 50% relative decrease in the frequency of I-*Sce*I-induced GCRs in clone EDS-7F2 (Table 3) when combining the eight experiments conducted for Figure 3B and D. Although the GCR assay showed a high standard deviation at interstitial DSBs due to the very low frequency of GCRs, the decrease in GCRs caused by Mirin in clone EDS-7F2 was consistently observed in four different experiments, each done in triplicate (see Supplementary Figures S3 and S4, and Table S1 for raw data) and were statistically significant (Figure 3B and D). Our results therefore show that the 3′–5′ exonuclease activity of MRE11 is involved in the formation of GCRs at interstitial I-*Sce*I-induced DSBs to a much greater extent than large deletions, although processing by other nucleases is also likely to be involved.

Consistent with our earlier results (49), clone EDS-6J8 with one subtelomeric I-*Sce*I-induced DSB (in pEJ5-GFP) and one interstitial I-*Sce*I-induced DSB (in pDsRed-ISceI) had a much greater frequency of GCRs than clones with two interstitial DSBs, with an average of 0.086% (Table 2) when combining the nine experiments conducted for Figure 3C and E. The inhibition of MRE11 with Mirin caused an average 51% relative reduction in the frequency of GCRs in clone EDS-6J8 (Table 3) when combining the nine experiments conducted for Figure 3C and E. Despite the low frequency of GCRs, the decrease in GCRs caused by Mirin in clone EDS-6D1 was consistently observed in the different experiments (see Supplementary Figures S3 and S4, and Table S1 for raw data) and was statistically significant (Figure 3C and E). Our results therefore show that the 3′–5′ exonuclease activity of MRE11 is involved in the formation of approximately half of the GCRs at both interstitial and subtelomeric DSBs. These results are consistent with previous studies showing that GCRs in mammalian cells primarily occur through the A-NHEJ pathway (69–71), and involve the processing of DSBs by CtIP (58), which functions in combination with MRE11 in processing DSBs. Based on our evidence that the inhibition of the 3′–5′ exonuclease activity of MRE11 with Mirin influences the formation of GCRs, we propose that, similar to large deletions, GCRs occur through processing by MRE11. However, the fact that Mirin does not entirely inhibit the formation of GCRs, suggests that other nucleases are also involved in the formation of GCRs.

### The knockdown of MRE11 has no effect on the formation of GCRs at interstitial DSBs, but inhibits GCRs at subtelomeric DSBs

The role ATM and MRE11 in the formation of GCRs was investigated using shRNA-mediated knockdown. The extent of knockdown following stable integration of the shATM vector in clone EDS-7F2 was determined to be 89% by qPCR (Table 1), and 85% by western blot analysis (Figure 1B and Table 1). The extent of knockdown following stable integration of the shMRE11 vector in clone EDS-7F2 was determined to be 80% by qPCR (Table 1), and 85% by western blot analysis (Figure 1B and Table 1). Consistent with our earlier report (49), in clone EDS-7F2 with an interstitial DSB, neither the knockdown of ATM (Figure 3B) nor the knockdown of MRE11 (Figure 3D) had a significant effect on the frequency of I-*Sce*I-induced GCRs. Although there were again large standard deviations, the results were consistently seen in different experiments (see Supplementary Figures S3 and S4 and Table S1 for raw data) and the results were significant (Figure 3B and D). Therefore, the effect of knockdown of MRE11 is very different from the inhibition of MRE11 with Mirin, in that it does not result in a decrease in the frequency of GCRs at interstitial DSBs. Although knockdown of MRE11 would inhibit MRE11 3′–5′ exonuclease activity similar to Mirin, the loss of other MRE11 functions following knockdown of MRE11 appears to compensate for the decrease in GCRs caused by the loss of MRE11 3′–5′ exonuclease activity. This may be due to an increase in processing of DSBs by other nucleases due to a loss of protection of DSBs in MRE11-deficient cells, because the knockdown of MRE11 has the same effect as knockdown of ATM in promoting GCRs.

The extent of knockdown by shATM in clone EDS-6J8 was determined to be 71% by qPCR (Table 1), and 77% by western blot analysis (Figure 1B and Table 1). The extent of knockdown following stable integration of the shMRE11 vector in clone EDS-6J8 was determined to be 80% by qPCR (Table 1), and 77% by western blot analysis (Figure 1B and Table 1). As we have previously shown (49), in clone EDS-6J8 with a subtelomeric DSB, the knockdown of ATM caused a 44% relative decrease in the frequency of I-*Sce*I-induced GCRs (Figure 3C). Similarly, the knockdown of MRE11 in clone EDS-6J8 caused a 52% relative decrease in the frequency of GCRs (Figure 3E). Although there were again large standard deviations, the results were consistently seen in different experiments (see Supplementary Figures S3 and S4 and Table S1 for raw data) and the results were significant (Figure 3C and E). Therefore, as with large deletions, the effect of knockdown of ATM on the frequency of GCRs is nearly identical to the effect of knockdown of MRE11 in both clone EDS-7F2 and clone EDS-6J8.

Importantly, the effect of knockdown of ATM and MRE11 on our assays is very different for large deletions and GCRs. The knockdown of MRE11 or ATM caused a much larger increase in the frequency of large deletions at interstitial DSBs than at subtelomeric DSBs (Figure 2). However, with GCRs, the knockdown of MRE11 or ATM had little or no effect on the frequency of GCRs at interstitial DSBs (Figure 3B and D), and inhibited GCRs at subtelomeric DSBs (Figure 3C and E). The fact that knockdown of MRE11 or ATM had no effect on GCRs at interstitial DSBs, but decreased the frequency of GCRs at subtelomeric DSBs shows that the role of ATM activation in preventing GCRs is very different at interstitial and subtelomeric DSBs. The difference in the effect of knockdown of ATM or MRE11 on large deletions and GCRs at interstitial and subtelomeric DSBs also demonstrates that GCRs can occur through a different mechanism than large deletions. Although large deletions near telomeres will often result in loss of the telomere, and therefore result in GCRs, we have previously demonstrated that GCRs near telomeres can also occur independently of large deletions (38). GCRs that occur in combination with large deletions are not detected by our GCR assay, and as a result, the frequency of GCRs detected by our assay will be increased by factors that promote processing without extensive resection, and decreased by factors that promote large deletions. The processing involved in the formation of GCRs can occur without extensive resection, as shown by the increase A-NHEJ in cells deficient in BRCA1, which have a deficiency in HRR (22), and by an fact that MRE11 is involved in A-NHEJ involving less than 20 bp of end resection (23).

### The inhibition of MRE11 has little or no effect on the formation of small deletions at either interstitial or subtelomeric DSBs

**Figure 4.**
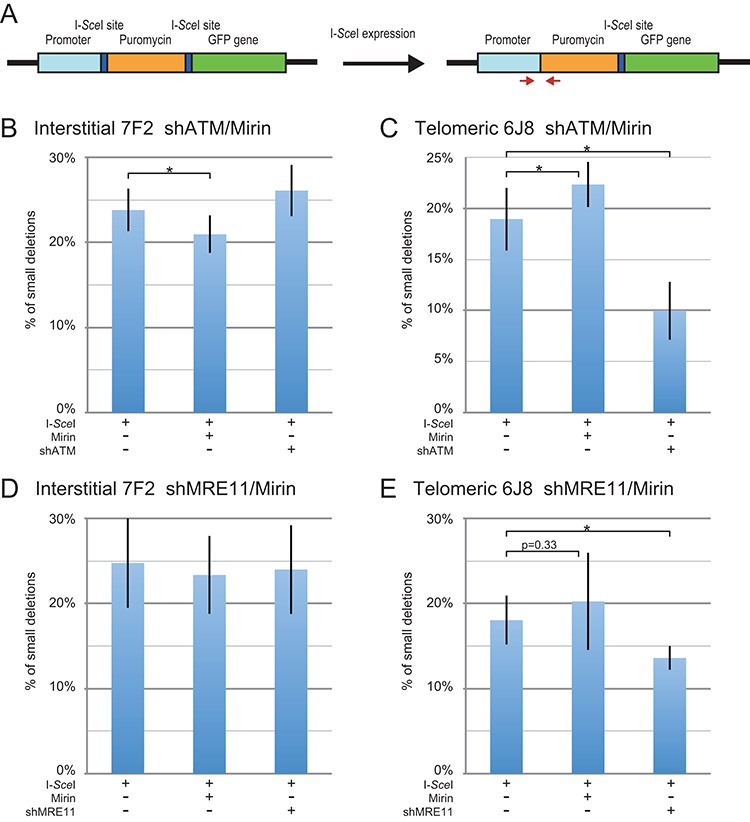
The effect of inhibition of MRE11 and ATM on small deletions at interstitial and subtelomeric DSBs. (**A**) Cell clones containing the pEJ5-GFP plasmid integrated at an interstitial (EDS-7F2) or telomeric (EDS-6J8) site were used for analysis of small deletions. Small deletions were determined by first amplifying a PCR product that spans one of the I-*Sce*I endonuclease recognition sites from genomic DNA isolated from the pooled population of cells expressing I-*Sce*I endonuclease. The PCR product was then digested with I-*Sce*I endonuclease to determine the frequency of cells in the population with small deletions at the I-*Sce*I-induced DSB, as shown by the fraction of PCR product that is not cut with I-*Sce*I endonuclease. The frequency of small deletions at the I-*Sce*I-induced DSB was determined for clone EDS-7F2 (**B, D**) and clone EDS-6J8 (**C, E**) following infection with the pQCXIH-ISceI retrovirus vector and selection with hygromycin for 14 days for EDS-7F2 and 15 days for EDS-6J8. Small deletions were analyzed following (**B, C**) treatment with Mirin or knockdown of ATM (shATM), or (**D, E**) treatment with Mirin or knockdown of MRE11 (shMRE11). Control cultures for knockdown of ATM or MRE11 were treated with shRNA-mediated knockdown of luciferase, while control cultures for Mirin were treated with DMSO. The values shown in the graph represent the average of more than three independent experiments, each done in triplicate. Error bars represent the standard deviation of more than three separate experiments. Statistical significance for comparisons between the indicated values (horizontal lines) was determined using the two-tailed Student's *t*-test, and an asterisk indicates statistically significant values of 0.05 or less.

We next analyzed whether MRE11 is involved in the formation of small deletions at interstitial or subtelomeric DSBs. The small deletion assay monitors the frequency of loss of a few nucleotides during NHEJ at a single I-*Sce*I-induced DSB in the pEJ5-GFP plasmid (Figure 4A). Small deletions are the most common I-*Sce*I-induced mutation at interstitial sites (43–45,64). In the study by Honma *et al*., 29 of 926 clones selected at random had mutations at the I-*Sce*I site (43). Of these 29 mutants, 19 were small deletions of 27 base pairs or less, nearly all of which were 9 base pairs or less. Only 5 contained large deletions of 60 bp or more. Our large deletion assay detects deletions of 29 bp or larger on one side of the DSB, while our small deletion assay monitors the loss of even a single base pair at the I-*Sce*I site.

In clone EDS-7F2 with an interstitial DSB, the frequency of I-*Sce*I-induced small deletions averaged 24.2% (Table 2) when combining the seven experiments shown for Figure 4B and D. The inhibition of MRE11 with Mirin slightly decreased the relative frequency of I-*Sce*I-induced small deletions at interstitial DSBs by an average of 9.4% (Table 3) when combining the seven experiments conducted for Figure 4B and D. Therefore, at interstitial DSBs, inhibition of the 3′–5′ exonuclease activity of MRE11 inhibited the formation of small deletions much less (9.4%) than it did large deletions (19%) or GCRs (50%).

In clone EDS-6J8 with a subtelomeric DSB, the frequency of I-*Sce*I-induced small deletions was similar to that in clone EDS-7F2, with an average of 18.5% (Table 2) when combining the seven experiments conducted for Figure 4C and E. Similar frequencies of small deletions have also been observed at I-*Sce*I-induced DSBs in clones GFP-7F1 and GFP-6D1, which contain a single I-*Sce*I site (Supplementary Figure S5). Therefore, unlike our large deletions assay, which shows a much greater frequency of large deletions at subtelomeric DSBs, our small deletion assay demonstrates little difference in the frequency of small deletions at interstitial and subtelomeric DSBs (42,49).

In clone EDS-6J8, Mirin caused a slight relative increase in the frequency of small deletions, averaging 16% when combining the seven experiments conducted for Figure 4C and E (Table 3), possibly due to the corresponding decrease in large deletions. Therefore, unlike large deletions and GCRs, the 3′–5′ exonuclease activity of MRE11 has a very minor role in the formation of small deletions at interstitial DSBs and no role in small deletions at subtelomeric DSBs. Combined with our results that, unlike large deletions and GCRs, small deletions are not increased at subtelomeric DSBs, our evidence suggests that small deletions occur through a different mechanism than large deletions and GCRs. Based on the fact that MRE11 is not involved in the formation of most small deletions, we propose that unlike large deletions and GCRs, most small deletions occur through the KU70/86-dependent C-NHEJ pathway.

### The frequency of small deletions is the same at interstitial and subtelomeric DSBs

An important result of our study is that unlike large deletions and GCRs, which are greatly increased at subtelomeric DSBs, the frequency of small deletions is similar at interstitial and subtelomeric DSBs. The average frequency of small deletions at subtelomeric DSBs in clone EDS-6J8 in the seven experiments performed for Figure 4C and E was 77% of the frequency of small deletions at interstitial DSBs in clone EDS-7F2 (Table 2). Moreover, when corrected for the much greater frequency of loss of the plasmid sequences as result of DSBs near telomeres, the rate of formation of small deletions is nearly identical at interstitial and subtelomeric DSBs (Table 2). This difference in large and small deletions is independent of the integration site, the plasmid used, or the type of selection, since we have observed similar results using other cell clones with different plasmids, with or without selection (40). Furthermore, the frequency of small deletions is very similar in clones GFP-7F1 and GFP-6D1, which contain a single I-*Sce*I site (Supplementary Figure S5). This correction for large deletions is necessary because although the rate of production of DSBs would initially be the same at interstitial and subtelomeric sites when I-*Sce*I endonuclease is first expressed, the higher rate of loss of the plasmid at telomeric sites due to large deletions would result in the gradual decline of the rate of production of DSBs in clone EDS-6J8. By the end of the experiment, 51% of the cells in clone EDS-6J8 would have lost the telomeric plasmid due to large deletions, and therefore the rate of formation of DSBs would be half of that observed in clone EDS-7F2. Therefore, over the course of the experiment, the average rate of formation of DSBs in clone EDS-6J8 would be 25.5% less than in clone EDS-7F2. The percentage of small deletions, as well as the frequency of large deletions, GCRs, and distal NHEJ must therefore be adjusted accordingly to compensate for the loss of the plasmid (Table 2). In view of our evidence that small deletions occur by C-NHEJ, the similar frequencies of small deletions at interstitial and subtelomeric DSBs suggests that C-NHEJ is fully functional near telomeres.

### The knockdown of MRE11 has minimal effect on the formation of small deletions at interstitial DSBs, but inhibits small deletions at subtelomeric DSBs

Consistent with our previous report (49), in clone EDS-7F2 with an interstitial DSB, the knockdown of ATM caused only a slight increase in the frequency of small deletions (Figure 4B), while in clone EDS-6J8 with a subtelomeric DSB, the knockdown of ATM caused a 47% relative decrease in the frequency of small deletions (Figure 4C). Similarly, MRE11 knockdown had little if any affect on the frequency of small deletions in clone EDS-7F2 (Figure 4D), while the knockdown of MRE11 caused a 25% decrease in the frequency of small deletions in clone EDS-6J8 (Figure 4E). The knockdown of ATM and Mirin had similar effects on the frequency of small deletions in clones GFP-7F1 and GFP-6D1, which contain a single I-*Sce*I site (Supplementary Figure S5). Therefore, unlike small deletions at interstitial DSBs, the formation of small deletions at subtelomeric DSBs is dependent on ATM, which is consistent with our conclusions above that small deletions occur by C-NHEJ, and that the repair of subtelomeric DSBs by C-NHEJ is ATM dependent.

### MRE11 is involved in distal NHEJ at interstitial DSBs, but not at subtelomeric DSBs

**Figure 5.**
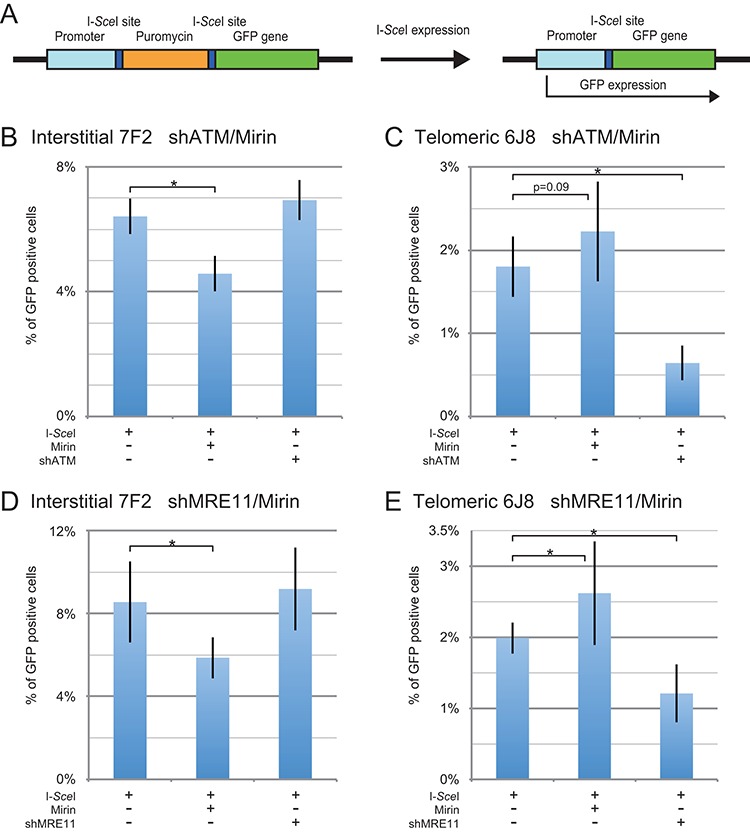
The effect of inhibition of MRE11 and ATM on distal NHEJ at interstitial and subtelomeric DSBs. (**A**) Cell clones containing the pEJ5-GFP plasmid integrated at an interstitial (EDS-7F2) or telomeric (EDS-6J8) site were used for analysis of distal NHEJ. The GFP gene in the pEJ5-GFP plasmid is initially inactive due to the presence of puromycin-resistance (puro) gene located between the GFP gene and its promoter, but is activated following NHEJ between the distal ends of the two I-*Sce*I-induced DSBs, which results in the deletion of the puro gene. The frequency of distal NHEJ (GFP-positive cells) at the I-*Sce*I-induced DSB was determined for clone EDS-7F2 (**B, D**) and clone EDS-6J8 (**C, E**) following infection with the pQCXIH-ISceI retrovirus vector and selection with hygromycin for 14 days for EDS-7F2 and 15 days for EDS-6J8. Distal NHEJ was analyzed following (**B, C**) treatment with Mirin or knockdown of ATM (shATM), or (**D, E**) treatment with Mirin or knockdown of MRE11 (shMRE11). Control cultures for knockdown of ATM or MRE11 were treated with shRNA-mediated knockdown of luciferase, while control cultures for Mirin were treated with DMSO. The values shown in the graph represent the average of the more than three independent experiments, each done in triplicate. Error bars represent the standard deviation of more than three separate experiments. Statistical significance for comparisons between the indicated values (horizontal lines) was determined using the two-tailed Student's *t*-test, and an asterisk indicates statistically significant values of 0.05 or less.

The role of MRE11 in the formation of deletions caused by the mis-joining of the distal ends of two closely positioned DSBs was determined using the distal NHEJ assay. The distal NHEJ assay monitors the activation of the GFP gene in a pEJ5-GFP plasmid that is integrated at either an interstitial or telomeric site (Figure 5A). The GFP gene in pEJ5-GFP is initially inactive due to the presence of a puro gene that is positioned between the GFP gene and its promoter. However the GFP gene can be activated by joining together the distal ends of two I-*Sce*I-induced DSBs, which are located 1.8 kb apart at either end of the puro gene. The activation of the GFP gene in the distal NHEJ assay can result from C-NHEJ or A-NHEJ (26), and can therefore be affected by changes in the KU70/86 pathway or the MRN/ATM pathway. As with the GCR assay, the distal NHEJ assay is also selective in that it does not detect repair events involving large deletions due to the loss of the GFP gene or its promoter.

The frequency of I-*Sce*I-induced distal NHEJ in clone EDS-7F2 with an interstitial DSB averaged 7.8% (Table 2) when combining the eight experiments conducted for Figure 5B and D. The inhibition of MRE11 with Mirin caused an average 32% relative decrease in the frequency of distal NHEJ in clone EDS-7F2 (Table 3) when combining the eight experiments conducted for Figure 5B and D. Therefore, our results suggest that the 3′–5′ exonuclease activity of MRE11 is involved in some of the distal NHEJ at interstitial DSBs. This result is consistent with an earlier study that found that Mirin was capable of partially inhibiting distal NHEJ in human and hamster cells (11).

In clone EDS-6J8 with a subtelomeric DSB, the frequency of distal NHEJ is much lower than in clone EDS-7F2, with an average of 2.0% (Table 2) when combining the seven experiments conducted for Figure 5C and E. The inhibition of MRE11 with Mirin in clone EDS-6J8 caused an average 23% relative increase in the frequency of distal NHEJ (Table 3) when combining the seven experiments conducted for Figure 5C and E, possibly due to the corresponding decrease in large deletions. Therefore, our results suggest that unlike at interstitial DSBs, the inhibition of the 3′–5′ exonuclease activity of MRE11 with Mirin does not inhibit, and even slightly increases, distal NHEJ at subtelomeric DSBs, demonstrating a fundamental difference in the repair pathways involved in distal NHEJ at interstitial and subtelomeric DSBs. In view of the absence of a role for MRE11 3′–5′ exonuclease activity, and therefore the processing of the DSBs, we propose that distal NHEJ at subtelomeric DSBs occurs primarily through the KU70/86-dependent pathway.

### The knockdown of MRE11 has no effect on distal NHEJ at interstitial DSBs, but inhibits distal NHEJ at subtelomeric DSBs

Consistent with our earlier report (49), the knockdown of ATM in clone EDS-7F2 with an interstitial DSB caused a small but not statistically significant increase in the frequency of distal NHEJ (Figure 5B). Similar to the knockdown of ATM, the knockdown of MRE11 in clone EDS-7F2 also showed a small but not statistically significant increase in the frequency of distal NHEJ (Figure 5D). Therefore, the knockdown of MRE11 or ATM is very different from the effect of inhibition of MRE11 3′–5′ exonuclease activity with Mirin, which caused a decrease in the frequency of distal NHEJ. In this regard, distal NHEJ behaves similarly to GCRs, suggesting that the loss of activation of ATM caused by knockdown of MRE11 results in the loss of end protection, which counteracts the loss of MRE11 3′–5′ exonuclease activity.

As we have previously reported (49), unlike with clone EDS-7F2, the knockdown of ATM in clone EDS-6J8 caused a large, 64% (from 1.8% to 0.6%), relative decrease in the frequency of distal NHEJ (Figure 5C). Similar to the knockdown of ATM, the knockdown of MRE11 caused a 39% (from 2.0% to 1.2%) relative decrease in the frequency of distal NHEJ at subtelomeric DSBs (Figure 5E). Therefore, unlike at interstitial DSBs, at subtelomeric DSBs the knockdown of MRE11 or ATM results in a substantial decrease in the frequency of distal NHEJ similar to that seen for GCRs, indicating a difference in the role of ATM activation in the processing of interstitial and subtelomeric DSBs.

## DISCUSSION

### A model for the mechanism of formation of mutations at DSBs

**Figure 6.**
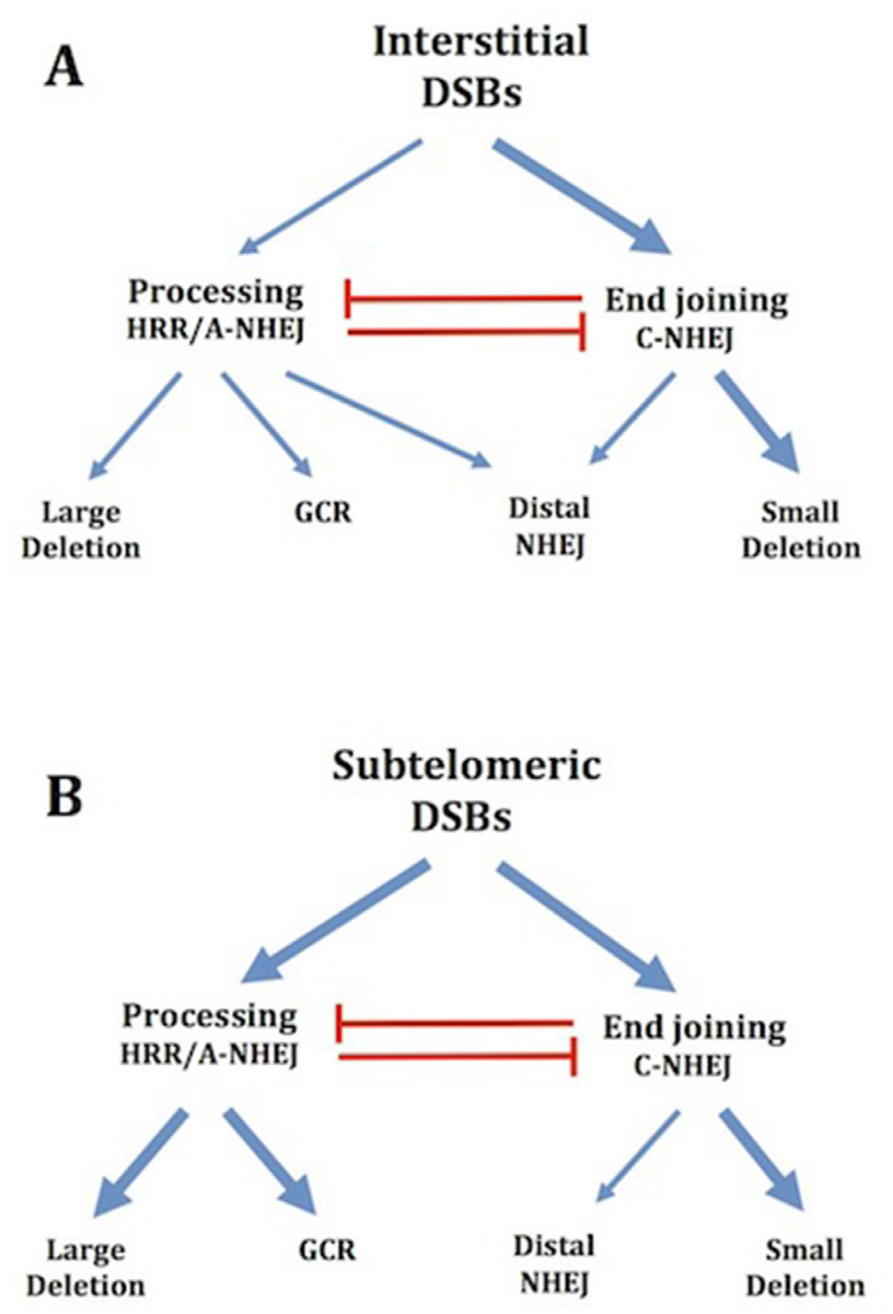
Model for the mechanisms of formation of mutations during repair of interstitial and subtelomeric I-*Sce*I-induced DSBs. **(A)** Mechanisms of formation of mutations at interstitial DSBs. DSB repair occurs either directly through C-NHEJ, or following the processing of the ends of the DSB. As with HRR, large deletions and GCRs also involve the processing of DSBs, however repair occurs by A-NHEJ. Importantly, the GCR assay does not detect GCRs that also involve large deletions. However, large deletions near telomeres also commonly result in GCRs, so that the major difference between the large deletion and GCR assays is the extent of degradation involved in the GCR. Small deletions of a few base pairs occur during end joining involving C-NHEJ. Distal NHEJ (deletions resulting from joining two closely positioned DSBs) occurs both by end joining by C-NHEJ and following processing and A-NHEJ. **(B)** Mechanisms of formation of mutations at subtelomeric DSBs. End joining by C-NHEJ at subtelomeric DSBs occurs with the same efficiency as at interstitial DSBs, as shown by the fact that small deletions at subtelomeric DSBs occur at the same frequency as at interstitial DSBs. The repair of subtelomeric DSBs by end joining by C-NHEJ is ATM-dependent. As at interstitial DSBs, large deletions and GCRs occur through processing of DSBs and A-NHEJ, although with a much greater frequency than at interstitial DSBs. The decreased frequency of distal NHEJ at subtelomeric DSBs appears to be due to a reduced contribution of A-NHEJ, possibly because most DSBs repaired by A-NHEJ at subtelomeric DSBs become large deletions and/or GCRs. Combined together, our results suggest that the sensitivity of subtelomeric regions to DSBs is a result of excessive processing by MRE11 and other nucleases, and is not due to a deficiency in C-NHEJ.

Based on our results, we now propose that large deletions and GCRs at both interstitial and subtelomeric DSBs are a result of excessive processing (Figure 6). Our results show that some of this excessive processing involves MRE11; however, the fact that Mirin only partially inhibits the formation of large deletions and GCRs suggests that other nucleases are also involved. This is expected from the fact that in addition to MRE11, other nucleases have been previously found to be involved in excessive processing at unprotected DSBs (4,5,58). Although large deletions and GCRs occur at both interstitial and subtelomeric DSBs, the much greater frequency of large deletions and GCRs at subtelomeric DSBs (49), and the much greater size of the large deletions at subtelomeric DSBs (40), demonstrates that subtelomeric DSBs are much more prone to excessive processing than interstitial DSBs. The involvement of MRE11 in the processing involved in the formation of large deletions and GCRs is consistent with the role of MRE11 in the processing of DSBs for HRR and A-NHEJ (8,9,11,12,26), and the involvement of A-NHEJ in large deletions and GCRs (19–21). The extensive deletions and microhomology at sites of chromosome fusions in human cells during crisis is also consistent with a role for excessive processing of DNA ends at unprotected telomeres (4,5,72).

Although the work here is limited to the analysis of clones of the EJ-30 bladder cell carcinoma cell line, the sensitivity of subtelomeric regions to DSBs has also been observed in other eukaryotic cells. Subtelomeric regions in yeast have been demonstrated to show greatly increased frequencies of GCRs in response to DSBs (73), and we have observed similar types of rearrangements in response to DSBs near telomeres in mouse embryonic stem cells (39). Moreover, persistent DSBs near telomeres have been shown to be a characteristic of normal human and rodent cells, both in culture and *in vivo* (36,37).

The differences in the frequencies of mutations at interstitial and subtelomeric DSBs cannot be explained by differences in selection, cell death, or the efficiency of generating DSBs with the I-*Sce*I endonuclease. This is clear from the different responses in the various assays, with the frequency of large deletions and GCRs being increased at subtelomeric DSBs, the frequency of distal NHEJ being decreased, and the frequency of small deletions being nearly identical at interstitial and subtelomeric DSBs. The frequency of HRR is also similar at interstitial and subtelomeric DSBs (42). This increased frequency of large deletions and GCRs at subtelomeric DSBs, and the similarity in the frequency of small deletions at interstitial and subtelomeric DSBs, has also been observed in our earlier studies using GFP expression (42,49) or selection with ganciclovir (40). We have also seen this same spectrum of mutations at interstitial and subtelomeric DSBs without selection, by randomly picking and analyzing 100 subclones for large deletions and small deletions following the introduction of I-*Sce*I-induced DSBs (40). The high frequency of large deletions and GCRs at subtelomeric DSBs has been seen with a variety of plasmids and other interstitial and telomeric sites, both in human tumor cells (38,40,67) and mouse embryonic stem cells (39,66).

As with any inhibitor, the use of Mirin in our studies brings up the possibility of off-target effects. As mentioned earlier, other studies have found that Mirin, and/or its derivative PFM39, are specific inhibitors of MRE11 3′–5′ exonuclease activity based on x-ray crystallography. Moreover, this study also showed that these inhibitors are specific for the 3′–5′ exonuclease activity of MRE11 in that they did not inhibit the activation of ATM, and had no effect on MRE11-deficient cells (7,63). Consistent with this specificity of Mirin for MRE11 3′–5′ exonuclease activity, we also found that Mirin did not inhibit the ability of MRE11 to activate ATM in response to ionizing radiation (Figure 1C).

Our results are consistent with previous studies that have demonstrated that MRE11 is involved in the degradation of the ends of DSBs leading to deletions and repair by A-NHEJ. In a study by Rahal *et al*. (24), Mirin was found to inhibit the degradation of the ends of a DSB in plasmid DNA added to cellular extracts, which, as in our study, was found to be mediated by MRE11 and was inhibited by ATM. As in our study, Mirin did not inhibit the activation of ATM in the study by Rahal *et al*. (24). However, for reasons that are not clear, the knockdown of MRE11 did not prevent the activation of ATM, as it does in live cells. In the study by Truong *et al*. (23), MRE11 was found to be involved in the repair of DSBs by microhomology mediated end joining (MMEJ, a form of A-NHEJ), using a plasmid containing a reporter gene that was integrated in mouse or human cells. The plasmid system used by Truong *et al*. detects deletions from 18 to 27 bp, which are diminished in cells with nuclease-deficient MRE11, as well as by the inhibition of CDK or CtIP (23). Therefore, A-NHEJ utilizes the same processing mechanism as HRR, as has been previously shown by the fact that A-NHEJ can occur in cells deficient in HRR (22), although A-NHEJ can occur with much less processing than HRR.

### Mechanism of formation of large deletions at interstitial and subtelomeric DSBs

An important observation in our study is that treatment with Mirin had the opposite effect of knockdown of MRE11 on the frequency of large deletions at both interstitial and subtelomeric DSBs. Although this may at first seem contradictory, it is entirely consistent with the multiple roles of MRE11 in DSB repair. Mirin specifically inhibits MRE11 3′–5′ exonuclease activity, which is ATM independent (10–12), thereby limiting the excessive processing of DSBs leading to large deletions. In contrast, knockdown of MRE11 prevents the activation of ATM, which is required for protection of DSBs (4), thereby promoting the excessive processing of DSBs. Proof that the effect of knockdown of MRE11 on large deletions is a result of a failure to activate ATM is shown by the fact that the effects of knockdown of MRE11 are nearly identical to the effects of knockdown of ATM (Figure 2) and the ATM kinase inhibitor KU55933 (49). Although knockdown of MRE11 would also inhibit MRE11 3′–5′ exonuclease activity, this would not inhibit the formation of large deletions, because the loss of protection would result in increased processing or resection by other nucleases, including EXO1, which is independent of ATM at I-*Sce*I-induced DSBs (15,31,32).

Although the knockdown of MRE11 or ATM causes a small increase in the frequency of large deletions at telomeric DSBs, this increase in large deletions is much less than that observed at interstitial DSBs. We conclude that this small increase in large deletions caused by knockdown of MRE11 or ATM at telomeric DSBs cannot be explained solely by the loss of protection of DSBs, because as discussed below, our results suggest that DSB protection is already compromised at subtelomeric DSBs. We therefore propose that the reason that knockdown of MRE11 or ATM promotes large deletions at subtelomeric DSBs is because ATM is required for repair of subtelomeric DSBs by C-NHEJ, possibly because subtelomeric regions are composed of heterochromatin (51), and ATM is required for repair of DSBs in heterochromatin (6,7). The requirement for ATM for C-NHEJ at subtelomeric DSBs is also consistent with our demonstration that the knockdown of ATM inhibits the formation of small deletions at subtelomeric DSBs, because as is discussed below, small deletions occur through the C-NHEJ pathway.

### Mechanism of formation of GCRs at interstitial and subtelomeric DSBs

Our results demonstrate that knockdown of MRE11 or ATM has little effect on the frequency of GCRs at interstitial DSBs, but causes a large decrease in the frequency of GCRs at subtelomeric DSBs. These results demonstrate that the processing involved in formation of the GCRs detected by our GCR assay is somehow different from the processing involved in the formation of large deletions. To understand the difference in response of GCRs and large deletions to knockdown of MRE11 or ATM, it is important to point out that the GCRs detected by our GCR assay are only those involving processing without extensive resection, because GCRs that occur in combination with large deletions are not detected by our GCR assay. Proof that GCRs can occur without extensive resection is provided by the observation that a deficiency in BRCA1, EXOI or BLM can promote A-NHEJ without promoting HRR (22,23). ATM can have very different roles in regulating processing and resection, as shown by the fact that the resection of I-*Sce*I-induced DSBs for HRR is independent of ATM and the nuclease activity of CtIP (15,31,32), while the processing of I-*Sce*I-induced DSBs by CtIP for A-NHEJ is ATM dependent. We therefore propose that at interstitial DSBs, any potential increase in the frequency of GCRs caused by the loss of ATM-dependent protection is canceled out by the loss of ATM-dependent processing. As a result, the failure to activate ATM has little effect on the frequency of GCRs detected by our assay, but causes an increase in the frequency of large deletions, which result from ATM-independent resection. In contrast, at subtelomeric DSBs, the decrease in the frequency of GCRs resulting from knockdown of MRE11 or ATM is because DSB protection is already compromised, which is the reason for the increased frequency of large deletions and GCRs at subtelomeric DSBs. As a result, any loss of ATM-dependent processing due to a failure to activate CtIP is not canceled out by a corresponding loss of protection, thereby resulting in a decrease in the GCRs detected by our assay. It is important to point out, however, that this only applies to GCRs that occur without large deletions, although the inhibition of ATM also results in an increased frequency of large deletions, which often involve GCRs.

The decrease in the frequency of GCRs at subtelomeric DSBs caused by knockdown of MRE11 or ATM could result from the corresponding increase in the frequency of large deletions. However, we do not favor this model, because the inhibition of ATM also causes an increase in large deletions at interstitial DSBs (Figure 2B), but no corresponding decrease in GCRs. In addition, ATM knockdown and inhibition of ATM with KU55933 are not additive in causing a decrease in the frequency of large deletions, but are additive for GCRs (49).

### Mechanism of formation of small deletions at interstitial and subtelomeric DSBs

Unlike with large deletions and GCRs, Mirin only slightly decreased the frequency of small deletions at interstitial DSBs, and increased the frequency of small deletions at subtelomeric DSBs. As a result, we propose that most small deletions occur through C-NHEJ, which does not require processing (Figure 6). This difference in the mechanism by which small deletions and large deletions/GCRs are formed explains why the frequency of small deletions is the same at interstitial and subtelomeric DSBs, even though the frequency of large deletions and GCRs is greatly increased at subtelomeric DSBs. Importantly, in view of the nearly identical frequency of small deletions at interstitial and subtelomeric DSBs, the conclusion that small deletions are formed during C-NHEJ means that subtelomeric regions are not deficient in C-NHEJ. Therefore, a deficiency in C-NHEJ cannot explain the sensitivity of subtelomeric regions to DSBs.

In contrast to interstitial DSBs, the knockdown of MRE11 or ATM causes a large decrease in the frequency of small deletions at subtelomeric DSBs. We propose that this decrease is due to the requirement for ATM for C-NHEJ near telomeres. As discussed earlier, this could be because subtelomeric regions are composed of heterochromatin, which requires ATM for repair of DSBs by C-NHEJ. This conclusion is consistent with the observation that the C-NHEJ involved in chromosome fusions resulting from telomere dysfunction is ATM dependent (68). The presence of heterochromatin at the subtelomeric region containing the integrated pGFP-ISceI plasmid in clone 6D1-GFP is suggested by the heterogeneity in expression of the GFP gene (Supplementary Figure S2), which is characteristic of telomere position effect (74,75).

### Mechanism of distal NHEJ at interstitial and subtelomeric DSBs

We previously concluded that subtelomeric regions are deficient in C-NHEJ, based on our observation that the frequency of distal NHEJ detected by our assay is much lower at subtelomeric DSBs than at interstitial DSBs (42). However, based on our analysis of small deletions, as mentioned above, we no longer conclude that there is a direct inhibition of C-NHEJ in subtelomeric regions. Instead, we now conclude that the sensitivity of subtelomeric regions to DSBs is due to excessive processing, which can indirectly inhibit C-NHEJ. This conclusion has caused us to reinterpret our data based on the distal NHEJ assay. In this regard, it is important to point out that although most I-*Sce*I-induced DSBs are repaired precisely (48), the distal NHEJ assay only detects a small minority of events that involve the mis-repair between the distal ends of two closely positioned DSBs. In addition, the activation of the GFP gene in the distal NHEJ assay can occur through either C-NHEJ or A-NHEJ (11,12,27,64,76), but cannot detect rearrangements in which excessive processing has occurred at either of the two DSBs. Therefore, other factors in addition to a deficiency in C-NHEJ, including increased processing or a decrease in A-NHEJ, can result in a decrease in the frequency in the distal NHEJ assay.

Importantly, our results show that Mirin, knockdown of MRE11, and knockdown of ATM, all have the opposite effect at subtelomeric DSBs than they do at interstitial DSBs. At subtelomeric DSBs, Mirin causes a modest increase in distal NHEJ, while knockdown of MRE11 or ATM causes a large decrease in distal NHEJ. Therefore, the 3′–5′ exonuclease activity of MRE11 is not required for distal NHEJ at subtelomeric DSBs, although MRE11 is required for activation of ATM. We propose that the best explanation for these results is that most distal NHEJ at interstitial DSBs in the EJ-30 tumor cell line occurs through A-NHEJ, which is inhibited by Mirin, while most distal NHEJ at subtelomeric DSBs occurs through C-NHEJ, which is not affected by Mirin, but is ATM dependent (Figure 6). Our conclusion that the 3′–5′ exonuclease activity of MRE11 is not required for C-NHEJ is consistent with earlier studies that concluded that MRE11 nuclease activity is involved in A-NHEJ, but not C-NHEJ (11,12,77). We also propose that the reason that A-NHEJ does not contribute to distal NHEJ at subtelomeric DSBs in our assay is because the processing involved in A-NHEJ at subtelomeric DSBs is too extensive to be detected by the distal NHEJ assay. Therefore, we conclude that the reason for the decrease in the frequency of distal NHEJ at subtelomeric DSBs is due to excessive processing during A-NHEJ, and is not due to a deficiency in C-NHEJ.

This interpretation would mean that large proportion of distal NHEJ at interstitial DSBs in the EJ-30 tumor cell line occurs by A-NHEJ. Other studies have found that the great majority of distal NHEJ at interstitial DSBs results from C-NHEJ (11,12,27,64,76), although one study found that A-NHEJ accounted for 44% of distal NHEJ (12). One explanation for the high contribution of A-NHEJ to distal NHEJ at interstitial DSBs in the EJ-30 tumor cell line used in our study is that the EJ-30 tumor cell line relies heavily on A-NHEJ for repair of DSBs, similar to many other human tumor cell lines (78–80).

The frequency of distal NHEJ at interstitial sites has been reported to be increased by knockdown of a number of proteins, including ATM, NBS1 and RAD50 in mouse ES cells, which has been suggested to result from either limiting the persistence of DSBs or promoting correct end tethering (81,82). In contrast, the knockdown of MRE11 has been found to inhibit distal NHEJ at interstitial DSBs in mouse ES cells (12) and in hamster and SV40-transformed human cells (11), which has been suggested to be due to its requirement for synapsis/tethering for C-NHEJ, or processing for A-NHEJ. Thus, the proteins of the MRN complex, as well as ATM, which is activated by the MRN complex, have been reported to either increase or decrease the frequency of distal NHEJ. Our results in clones of the EJ-30 human tumor cell line differ from these earlier studies in that we find that knockdown of ATM and MRE11 has no effect on distal NHEJ at interstitial DSBs. However, as in the study by Xie *et al*. (12), we did observe that Mirin inhibits distal NHEJ. It is not clear why the knockdown of ATM, NBS1, RAD50, and MRE11 can have such different effects on distal NHEJ. One possibility is the different types of cells being used in the various studies. Alternatively, it could be the chromosome location or the extent of transcription, since transcription has been shown to affect the frequency of distal NHEJ (82). Regardless, based on our results with Mirin, we agree with the conclusions of Rass *et al*. (11) and Xie *et al*. (12) that the nuclease activity of MRE11 mediates distal NHEJ through A-NHEJ.

### The mechanism responsible for excessive processing of DSBs near telomeres

We previously proposed that the sensitivity of subtelomeric regions to DSBs is associated with the presence of the telomeric protein TRF2 (83). This conclusion was based on our observation that telomeric repeat sequences located adjacent to an interstitial DSB resulted in a high frequency of large deletions and a reduced frequency of distal NHEJ (42). Our conclusion was subsequently supported by the demonstration that TRF2 tethered adjacent to an interstitial DSB could interfere with DSB repair (36). The combined subtelomeric regions that are sensitive to DSBs constitutes a significant part of the human genome, since we have previously shown that the region that is sensitive to DSBs extends at least 100 kb from the telomere (38). In fact, the subtelomeric region that is sensitive to DSBs may be even larger, because as we have previously pointed out (83), the studies demonstrating the persistence of IR-induced DSBs near telomeres used doses of IR that would require targets on the ends of chromosomes that are 168–396 kb in length in order to generate persistent DSBs at the reported frequency (36,37).

How TRF2 or other telomeric proteins might promote large deletions and GCRs in a cis-acting manner remains unclear. As discussed above, our results suggest that subtelomeric DSBs are poorly protected, consistent with study showing that unprotected telomeres are extensively processed by EXO1 and CtIP (4,5). As we previously pointed out, TRF2 could contribute to excessive processing of subtelomeric DSBs either through its ability to bind and inhibit ATM, or through its role in regulating the processing of the ends of chromosomes (49,83). The inhibition of ATM by TRF2 does not appear to be consistent with our results, in that ATM is clearly functional in telomeric regions, as shown by the fact that the inhibition of ATM causes a further increase in the frequency of large deletions and a decrease in the frequency of GCRs, small deletions, and distal NHEJ at subtelomeric DSBs. However, we cannot rule out the possibility that ATM is partially inhibited near telomeres (52). Alternatively, we have proposed that the excessive processing of subtelomeric DSBs is because they are mistaken for telomeres (83). The blunt-ended leading strand at the end of the chromosome must be processed following DNA replication, which is coordinated by TRF2, and involves the nuclease activity of Apollo (56,57), as well as EXOI (57) and MRE11 (53–55). This processing is regulated by the binding of POT1/TPP1 to the single-stranded overhang, and as a result, cells deficient in POT1/TPP1 show extensive processing with very long single-stranded overhangs (84,85) and chromosome fusions involving A-NHEJ (86). Because POT1/TPP1 would not bind the single-stranded DNA generated by TRF2-mediated processing of subtelomeric DSBs, this processing would continue unchecked, similar to telomeres in POT1/TPP1 deficient cells.

One important clue comes from our earlier demonstration that HRR functions normally at subtelomeric DSBs (42), which suggests that the processing and resection of subtelomeric DSBs is not dysfunctional in late S/early G2 phase when HRR occurs. The sensitivity of subtelomeric regions to DSBs might therefore result from a failure to prevent processing of DSBs in G1 phase when HRR is not functional, which would indirectly inhibit C-NHEJ because single-stranded overhangs cannot be rejoined by C-NHEJ. The excessive processing of subtelomeric DSBs during G1 phase would also be consistent with the persistence of IR-induced DSBs near telomeres in non-dividing cells (36,37), while we see that subtelomeric DSBs are rejoined by A-NHEJ in dividing cells. However, the excessive processing of DSBs in G1 would not appear to be consistent with a model in which DSBs are mistaken and processed like telomeres, since telomere processing occurs in late S phase (87). The mechanism responsible for the deficiency in protection and excessive processing of DSBs near telomeres therefore remains to be determined.

### Importance of the sensitivity of subtelomeric regions to DSBs in human genetics and disease

Regardless of the mechanism responsible, it is now clear that the sensitivity of subtelomeric regions to DSBs has important consequences for human evolution and disease. Subtelomeric regions are very dynamic and serve as a birthplace of new genes, while many human genetic diseases result from alterations near the ends of chromosomes (88,89). The persistent DSBs near telomeres in human fibroblasts in culture and rat astrocytes *in vivo* are also associated with radiation-induced senescence (36,37). Because of the severe impact that senescent cells can have on surrounding tissues due to the secretion of senescence-associated secretory phonotype (SASP) proteins (90), IR-induced senescence will have important consequences for the loss of tissue function during aging. The sensitivity of subtelomeric regions to DSBs is also important in cancer. As pointed out earlier, oncogene-induced replication stress results in telomere dysfunction, which may serve as a mechanism for preventing cancer in normal cells (91). However, the sensitivity of subtelomeric regions to DSBs may also serve as a mechanism for telomere loss and chromosome instability in cancer cells (41). The further investigation of the mechanism of sensitivity of subtelomeric regions to DSBs is therefore important for understanding human genetic disease, aging and cancer.

## Supplementary Material

SUPPLEMENTARY DATA
