# Supplementary material for: Processing by MRE11 is involved in the sensitivity of subtelomeric regions to DNA double-strand breaks
Source: Nucleic Acids Res. 2015 Jul 23;43(16):7911–30. doi: 10.1093/nar/gkv714 (PMC4652756; doi:10.1093/nar/gkv714)
Supplement: SUPPLEMENTARY DATA [file supp_43_16_7911__index.html]

Processing by MRE11 is involved in the sensitivity of subtelomeric regions to DNA double-strand breaks — Processing by MRE11 is involved in the sensitivity of subtelomeric regions to DNA double-strand breaks — SUPPLEMENTARY DATA 

# Processing by MRE11 is involved in the sensitivity of subtelomeric regions to DNA double-strand breaks

## SUPPLEMENTARY DATA

- SUPPLEMENTARY DATA
