# Supplementary material for: Processing by MRE11 is involved in the sensitivity of subtelomeric regions to DNA double-strand breaks
Source: Nucleic Acids Res. 2015 Jul 23;43(16):7911–30. doi: 10.1093/nar/gkv714 (PMC4652756; doi:10.1093/nar/gkv714)
Supplement: SUPPLEMENTARY DATA [file supp_gkv714_nar-00734-d-2015-File008.pdf]

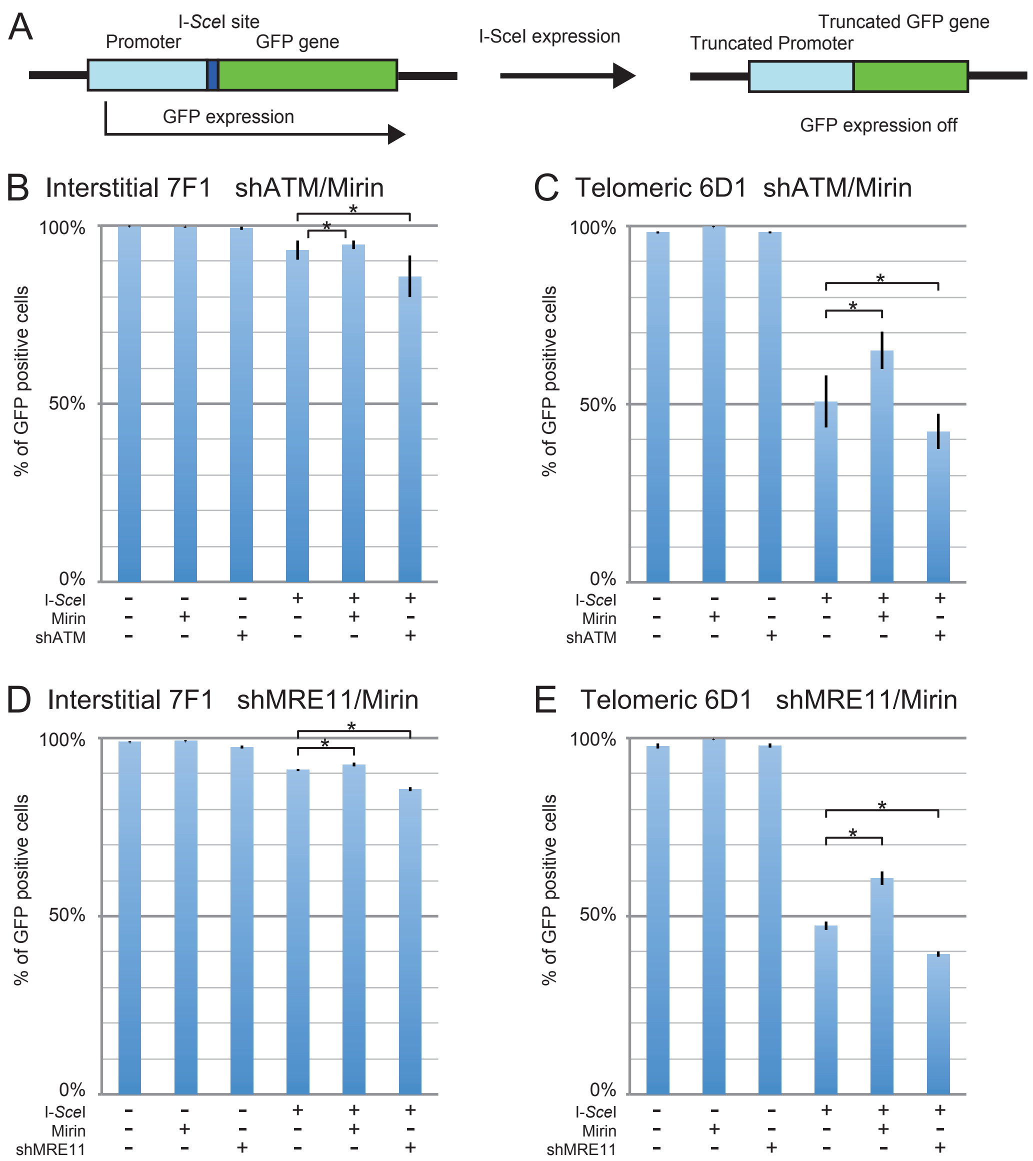

A
I-SceI site
Promoter
GFP gene
GFP expression
I-SceI expression
Truncated GFP gene
Truncated Promoter
GFP expression off
B Interstitial 7F1 shATM/Mirin
C Telomeric 6D1 shATM/Mirin
D Interstitial 7F1 shMRE11/Mirin
E Telomeric 6D1 shMRE11/Mirin
% of GFP positive cells
100%
50%
0%
I-SceI
Mirin
shATM
shMRE11
*

Figure S1

Figure S1. The effect of inhibition of MRE11 and ATM on large deletions at interstitial and subtelomeric DSBs. The results are the same as those shown in Figure 1, except that the data is plotted as GFP-positive cells. (**A**) Cell clones containing the pGFP-ISceI plasmid integrated at an interstitial (GFP-7F1) or telomeric (GFP-6D1) site were used for analysis of large deletions. The GFP gene in the integrated pGFP-ISceI plasmid is inactivated following large deletions of more than 28 bps at the I-*Sce*I-induced DSB. The frequency of large deletions (GFP-negative cells) in clone GFP-7F1 (**B, D**) and in clone GFP-6D1 (**C, E**) following infection with the pQCXIH or pQCXIH-ISceI retrovirus vector and selection with hygromycin for 14 days. Large deletions were analyzed following (**B, C**) treatment with Mirin or knockdown of ATM (shATM), or (**D, E**) treatment with Mirin or knockdown of MRE11 (shMRE11). Control cultures for knockdown of ATM or MRE11 were treated with shRNA-mediated knockdown of luciferase, while control cultures for Mirin were treated with DMSO. All samples were analyzed in triplicate. Error bars represent the standard deviation from three separate experiments. Statistical significance for comparisons between the indicated values (horizontal lines) was determined using the two-tailed Student's t-Test, and an asterisk indicates statistically significant values of 0.05 or less.

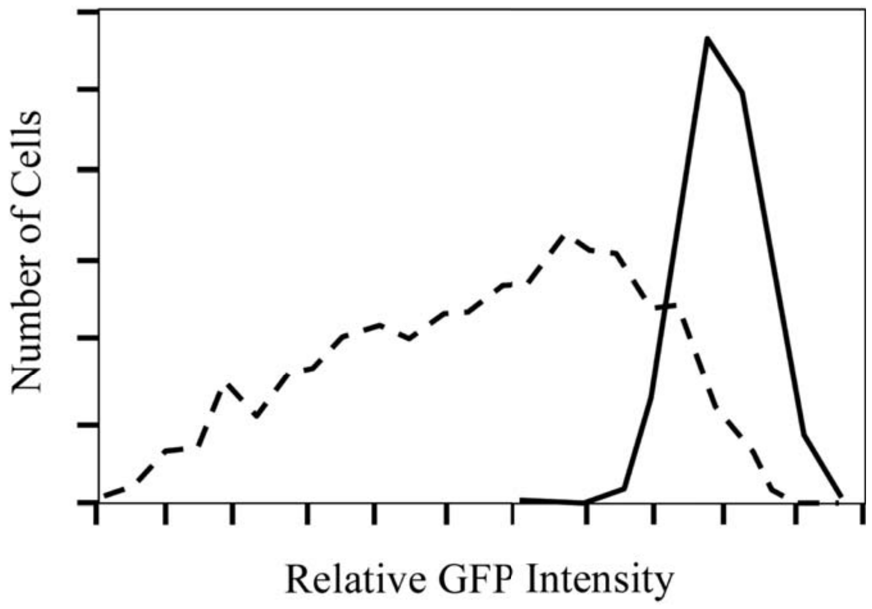

Figure S2

Figure S2. Relative expression of GFP in cells with interstitial and telomeric integration sites. The relative level of expression of GFP in clone GFP-7F1 (solid line) with an interstitial integration site is high and uniform in different cells in the population. In contrast, the level of expression of the GFP gene in clone GFP-6D1 (dashed line) with a telomeric integration site is lower and heterogeneous in different cells in the population, although almost all cells show detectable levels of expression. The distribution of the level of GFP expression was determined using a Cellometer (Nexelcom).

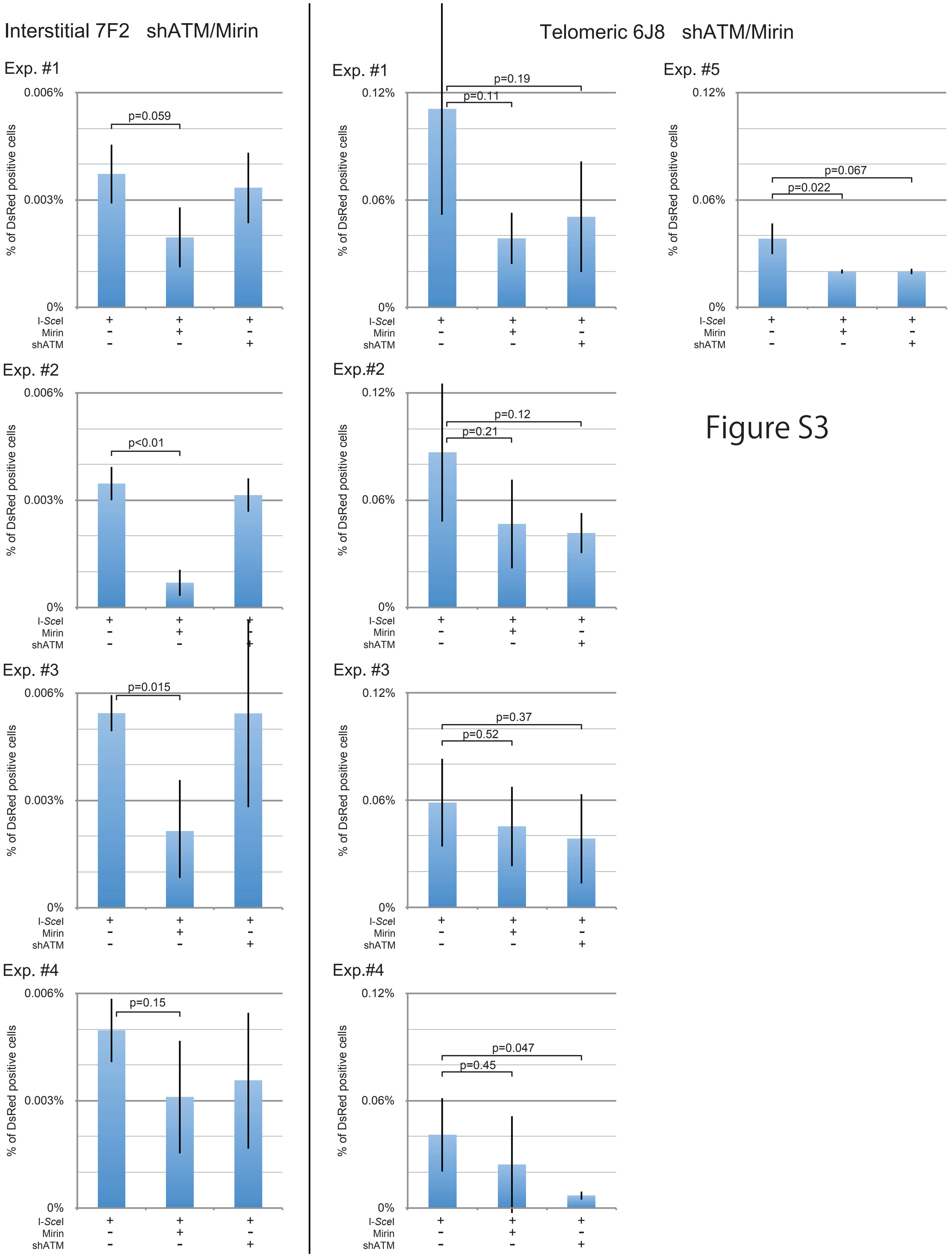
Interstitial 7F2 shATM/Mirin
Telomeric 6J8 shATM/Mirin
Exp. #1
Exp. #2
Exp. #3
Exp. #4
Exp. #5
Exp.#2
Exp.#4
% of DsRed positive cells
0.006%
0.003%
0%
0.12%
0.06%
I-SceI
Mirin
shATM
p=0.059
p<0.01
p=0.015
p=0.15
p=0.19
p=0.11
p=0.12
p=0.21
p=0.37
p=0.52
p=0.047
p=0.45
p=0.067
p=0.022

Figure S3

Figure S3. The individual experiments conducted to determine the effects of inhibition Mirin and knockdown of ATM on GCRs in clones EDS-7F2 and EDS-6J8. The frequency of GCRs at the I-*Sce*I-induced DSB was determined following infection with the pQCXIH-ISceI retrovirus vector and selection with hygromycin in four experiments with EDS-7F2 (Exp. #1-4) and five experiments with EDS-6J8 (Exp. #1-5), each done in triplicate. GCRs were analyzed following treatment with Mirin or knockdown of ATM (shATM). Control cultures for knockdown of ATM were treated with shRNA-mediated knockdown of luciferase, while control cultures for Mirin were treated with DMSO. The values shown in the graph represent the average of more than three independent experiments, each done in triplicate (see Table S1 for raw data). Error bars represent the standard deviation of the triplicate samples. Statistical significance for comparisons between the indicated values (horizontal lines) was determined using the two-tailed Student's t-Test, and an asterisk indicates statistically significant values of 0.05 or less.

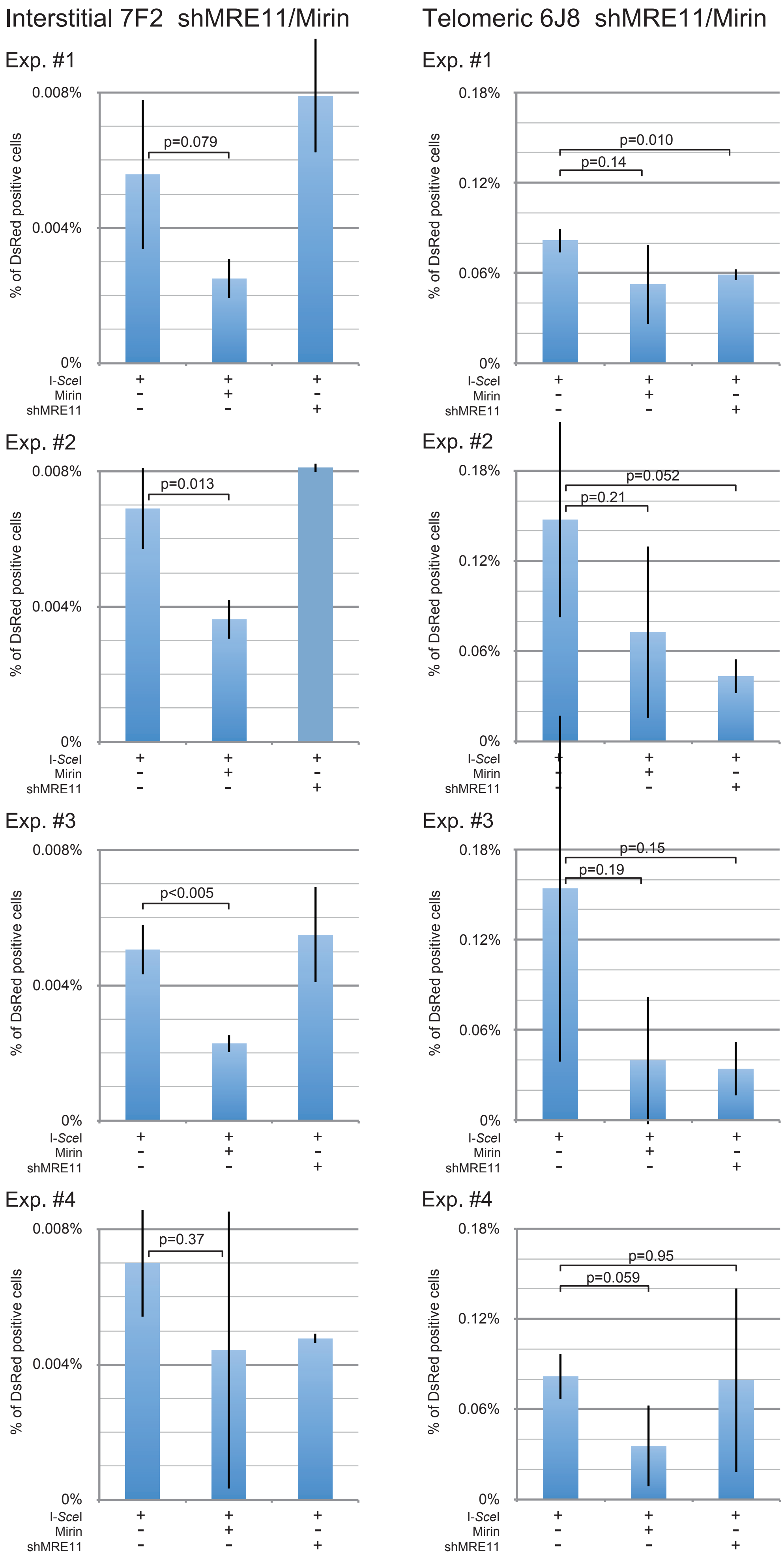
Interstitial 7F2 shMRE11/Mirin
Telomeric 6J8 shMRE11/Mirin
Exp. #1
Exp. #2
Exp. #3
Exp. #4
% of DsRed positive cells
0.008%
0.004%
0%
0.18%
0.12%
0.06%
I-SceI
Mirin
shMRE11
p=0.079
p=0.013
p<0.005
p=0.37
p=0.010
p=0.14
p=0.052
p=0.21
p=0.15
p=0.19
p=0.95
p=0.059

Figure S4

Figure S4. The individual experiments conducted to determine the effects of inhibition Mirin and knockdown of MRE11 on GCRs in clones EDS-7F2 and EDS-6J8. The frequency of GCRs at the I-*Sce*I-induced DSB was determined following infection with the pQCXIH-ISceI retrovirus vector and selection with hygromycin in four experiments with EDS-7F2 (Exp. #1-4) and four experiments with EDS-6J8 (Exp. #1-4), each done in triplicate. GCRs were analyzed following treatment with Mirin or knockdown of MRE11 (shMRE11). Control cultures for knockdown of MRE11 were treated with shRNA-mediated knockdown of luciferase, while control cultures for Mirin were treated with DMSO. The values shown in the graph represent the average of more than three independent experiments, each done in triplicate (see Table S1 for raw data). Error bars represent the standard deviation of the triplicate samples. Statistical significance for comparisons between the indicated values (horizontal lines) was determined using the two-tailed Student's t-Test, and an asterisk indicates statistically significant values of 0.05 or less.

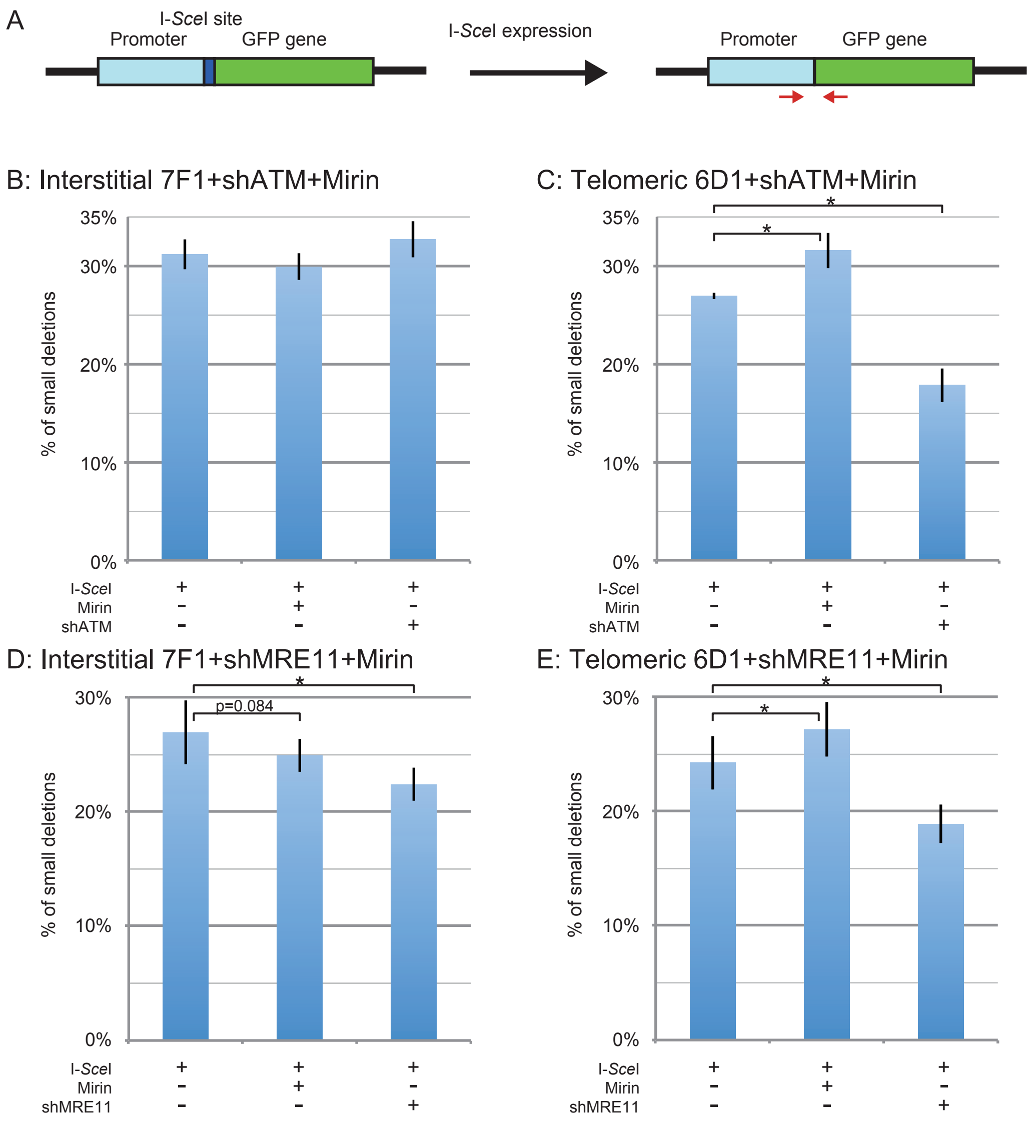
A
I-SceI site
Promoter
GFP gene
I-SceI expression
Promoter
GFP gene
B: Interstitial 7F1+shATM+Mirin
35%
30%
20%
10%
0%
% of small deletions
I-SceI
Mirin
shATM
C: Telomeric 6D1+shATM+Mirin
*
*
35%
30%
20%
10%
0%
% of small deletions
I-SceI
Mirin
shATM
D: Interstitial 7F1+shMRE11+Mirin
*
p=0.084
30%
20%
10%
0%
% of small deletions
I-SceI
Mirin
shMRE11
E: Telomeric 6D1+shMRE11+Mirin
*
*
30%
20%
10%
0%
% of small deletions
I-SceI
Mirin
shMRE11

Figure S5

Figure S5. The effect of inhibition of MRE11 and ATM on small deletions at interstitial and subtelomeric DSBs. (A) Cell clones containing the GFP-ISceI plasmid integrated at an interstitial (GFP-7F1) or telomeric (GFP-6D1) site were used for analysis of small deletions. Small deletions were determined by first amplifying a PCR product that spans one of the I-SceI endonuclease recognition sites from genomic DNA isolated from the pooled population of cells expressing I-SceI endonuclease. The PCR product was then digested with I-SceI endonuclease to determine the frequency of cells in the population with small deletions at the I-SceI-induced DSB, as shown by the fraction of PCR product that is not cut with I-SceI endonuclease. The frequency of small deletions at the I-SceI-induced DSB was determined for clone GFP-7F1 (B, D) and clone GFP-6D1 (C, E) following infection with the pQCXIH-ISceI retrovirus vector and selection with hygromycin for 14 days for GFP-7F1 and 15 days for GFP-6D1. Small deletions were analyzed following (B, C) treatment with Mirin or knockdown of ATM (shATM), or (D, E) treatment with Mirin or knockdown of MRE11 (shMRE11). Control cultures for knockdown of ATM or MRE11 were treated with shRNA-mediated knockdown of luciferase, while control cultures for Mirin were treated with DMSO. The values shown in the graph represent the average of more than three independent experiments, each done in triplicate. Error bars represent the standard deviation of more than three separate experiments. Statistical significance for comparisons between the indicated values (horizontal lines) was determined using the two-tailed Student's t-Test, and an asterisk indicates statistically significant values of 0.05 or less.

Fig. 3B

	7F2 shLuc/DMSO			7F2 shLuc/Mirin			7F2 shATM/DMSO		
	total # cells	# DsRed+	% DsRed+	total # cells	# DsRed+	% DsRed+	total # cells	# DsRed+	% DsRed+
exp #1	829,913	30	0.00361	413,925	11	0.00265	513,981	23	0.00447
	911,991	27	0.00296	554,526	12	0.00216	524,623	14	0.00266
	914,698	42	0.00459	484,916	5	0.00103	660,484	19	0.00287
exp #2	631,169	25	0.00396	642,308	2	0.00031	546,317	19	0.00347
	883,529	27	0.00305	546,051	4	0.00073	535,509	14	0.00261
	832,081	28	0.00336	578,290	6	0.00103	570,152	19	0.00333
exp #3	654,145	33	0.00504	221,828	7	0.00315	168,559	13	0.00771
	589,326	31	0.00526	229,244	6	0.00261	199,369	12	0.00601
	598,854	36	0.00601	300,948	2	0.00066	155,659	4	0.00256
exp #4	315,297	18	0.00571	189,196	9	0.00475	214,456	3	0.00139
	275,678	11	0.00399	123,654	2	0.00161	181,196	9	0.00496
	346,965	18	0.00518	102,028	3	0.00294	277,374	12	0.00432

Fig.3C

	6J8 shLuc/DMSO			6J8 shLuc/Mirin			6J8 shATM/DMSO		
	total # cells	# DsRed+	% DsRed+	total # cells	# DsRed+	% DsRed+	total # cells	# DsRed+	% DsRed+
exp #1	634,893	1127	0.17751	252,538	63	0.02494	471,925	401	0.08497
	806,078	731	0.09068	246,721	132	0.0535	474,568	198	0.04172
	831,613	536	0.06445	156,173	58	0.03713	457,980	115	0.02511
exp #2	691,486	740	0.10701	557,559	408	0.07317	488,600	217	0.04441
	933,752	1036	0.11095	740,187	320	0.04323	531,041	271	0.05103
	930,751	392	0.04211	664,720	158	0.02376	359,241	105	0.02922
exp #3	913,606	480	0.05253	739,215	454	0.06141	873,032	581	0.06654
	925,503	348	0.03762	810,683	442	0.05452	908,209	270	0.02972
	936,200	802	0.08566	785,678	156	0.01985	900,046	170	0.01888
exp #4	648,129	415	0.06403	134,417	21	0.01562	295,137	24	0.00813
	731,853	182	0.02486	158,685	4	0.00252	344,809	29	0.00841
	717,282	242	0.03373	96,790	53	0.05475	351,735	15	0.00426
exp #5	719,573	314	0.04363	636,824	120	0.01884	822,841	172	0.02092
	895,183	253	0.02826	717,071	142	0.01981	876,839	165	0.01881
	900,243	387	0.04298	774,977	165	0.02129			

Fig.3D

	7F2 shLuc/DMSO			7F2 shLuc/Mirin			7F2 shMRE11/DMSO		
	total # cells	# DsRed+	% DsRed+	total # cells	# DsRed+	% DsRed+	total # cells	# DsRed+	% DsRed+
exp #1	689,919	53	0.00768	125,873	3	0.00238	697,524	54	0.00774
	455,653	15	0.00329	149,939	3	0.00212	459,668	29	0.00631
	744,545	43	0.00577	160,518	5	0.00311	611,046	59	0.00965
exp #2	975,942	54	0.00553	975,121	30	0.00307	970,246	78	0.00803
	921,306	71	0.00771	975,106	41	0.00421	970,931	78	0.00803
	978,577	73	0.00745	977,337	35	0.00358	922,105	76	0.00824
exp #3	706,016	30	0.00424	232,925	6	0.00257	951,798	43	0.00451
	723,774	41	0.00566	187,257	4	0.00213	927,487	45	0.00485
	819,726	43	0.00524	281,859	6	0.00212	971,024	69	0.00713
exp #4	293,530	19	0.00647	38,510	2	0.00519	527,646	26	0.00492
	285,024	25	0.00877	12,182	0	0	423,853	20	0.00471
	243,616	14	0.00574	12,369	1	0.00808	514,290	24	0.00466

Fig. 3E

	6J8 shLuc/DMSO			6J8 shLuc/Mirin			6J8 shMRE11/DMSO		
	total # cells	# DsRed+	% DsRed+	total # cells	# DsRed+	% DsRed+	total # cells	# DsRed+	% DsRed+
exp #1	893,375	680	0.07611	114,919	95	0.08266	939,549	521	0.05545
	944,112	856	0.09066	121,118	49	0.04045	905,112	534	0.05899
	966,971	755	0.07807	113,711	39	0.03429	844,682	527	0.06239
exp #2	817,123	1628	0.19923	187,300	214	0.11425	820,721	286	0.03484
	344,658	257	0.07456	129,582	10	0.00771	378,869	212	0.05595
	435,619	736	0.16895	156,669	151	0.09638	376,304	148	0.03932
exp #3	310,135	296	0.09544	62,549	3	0.00479	792,814	403	0.05083
	417,347	349	0.08362	62,927	31	0.04926	748,927	1116	0.14901
	414,895	274	0.06604	68,148	36	0.05281	949,080	358	0.03772
exp #4	202,165	476	0.23545	59,279	9	0.01518	348,428	74	0.02123
	257,769	187	0.07254	53,079	47	0.08854	398,496	107	0.02685
				39,521	6	0.01518	377,275	206	0.05461

Table S1. Raw data for the frequency of DsRed-positive cells (DsRed+) obtained by FACs analysis of GCRs. The raw data is shown for both clone EDS-7F2 with an interstitial DSB (3B, 3D), and clone EDS-6J8 with a subtelomeric DSB (3C, 3E). Results are shown for cultures (7F2 shLuc/DMSO, 6J8 shLuc/DMSO), cultures treated with Mirin (7F2 shLuc/Mirin, 6J8 shLuc/Mirin), cultures with knockdown of ATM (7F2 shATM/DMSO, 6J8 shATM/DMSO, and cultures with knockdown of MRE11 (7F2 shMRE11/DMSO, 6J8 shMRE11/DMSO). Columns provide numbers for the total number of cells counted (total # cells), the number of DsRed-positive cells (# DsRed+), and the percent of DsRed-positive cells in the population counted (% DsRed+). The values are listed for each of the independent experiments (each done in triplicate) repeated for the figures, (3B) four experiments (exp #1 to #4), (3C) five experiments (exp #1 to #5), (3D) four experiments (exp #1 to #4), and (3E) four experiments (exp #1 to #4).
